# Management of Tarlov cysts: an uncommon but potentially serious spinal column disease—review of the literature and experience with over 1000 referrals

**DOI:** 10.1007/s00234-023-03226-6

**Published:** 2023-10-13

**Authors:** Kieran Murphy, Mehran Nasralla, Gaylene Pron, Khaled Almohaimede, Wouter Schievink

**Affiliations:** 1 Department of Medical Imaging, Toronto Western Hospital, University Health Network, 399 Bathurst St, Toronto, ON M5T 2S8 Canada; 2 Dalla Lana School of Public Health, Institute Health Policy, Management and Evaluation, University of Toronto, 155 College Street, Toronto, ON M5T 3M7 Canada; 3 Department of Neurosurgery, Cedars-Sinai Medical Center, 127 South San Vicente Boulevard, 6Th Floor, Los Angeles, CA 90048 USA

**Keywords:** Tarlov cysts, Cerebral spinal fluid, Coccygodynia, Fibrin sealant, Magnetic resonance imaging, Sacral dermatomes

## Abstract

Tarlov cysts were thought to be anatomic variants of uncertain etiology and clinical significance when initially described over 80 years ago. They are often detected in routine lumbosacral imaging and generally not reported in a differential diagnosis. There is increasing evidence that at least some Tarlov cysts are symptomatic and can have a significant adverse impact on patients’ health and well-being. Women are disproportionately affected with this condition, often presenting with long-standing pain and neurological dysfunctions. Significant gender bias has been a concern in the management of these patients. Unfortunately, there is no consensus on patient selection or management approaches for symptomatic Tarlov cysts. This review article updates information on the prevalence, diagnosis, clinical significance, and treatments of these cysts. Based on these findings and experience with over 1000 patient referrals, a treatment decision algorithm for symptomatic Tarlov cysts was constructed to provide guidance for appropriate management of patients with these complex cysts.

## Introduction

In 1938, Isadore Max Tarlov, a McGill University neurosurgeon in Montreal, Quebec, Canada, first characterized perineural cysts of the sacral roots that bear his name in cadavers that he dissected at the University [1]. He initially saw them as anatomic variants of uncertain etiology and clinical significance. Tarlov’s papers were widely read, and his initial assumptions about uncertain clinical significance of these cysts have persisted.

This review article updates information on the prevalence, diagnosis, clinical significance, and treatment of Tarlov cysts. There is no consensus on the management of these cysts, and based on these findings and experience with over 1000 patient referrals, a treatment decision algorithm for symptomatic Tarlov cysts was constructed to provide guidance for appropriate management of patients with these complex cysts.

## Anatomy and pathogenesis

There are numerous benign cystic conditions that can involve the spine and spinal cord. Since Tarlov [2] first detailed characteristics of cystic lesions of the spinal nerve roots and introduced the term perineural cysts, various other spinal cyst-like abnormalities have been identified and an array of confusing terms have been employed. Goyal et al. [3] first introduced a simplified classification for the various intraspinal conditions differentiating perineural cysts, root sleeve dilations, intradural or extradural arachnoid cysts, and meningeal diverticulum. Since then, Nabors et al. [4] described spinal meningeal cysts as diverticula of the spinal meningeal sac, nerve root sheath, or arachnoid. For simplicity, he referred to them all as meningeal cysts and introduced a simplified MR-based classification for these conditions consisting of three groups—type I, extradural meningeal cysts without spinal nerve root fibers; type II, extradural meningeal cysts with spinal nerve root fibers; and type III, spinal intradural meningeal cysts.

**Fig. 1:**
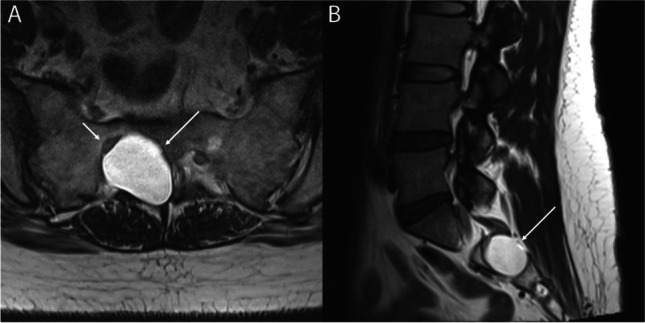
T2-weighted images of a Tarlov cyst. **A** Axial images shows the cyst expanding and remodeling the right S2 foramen and sacral canal (arrows) with the S2 nerve displaced and flattened (short arrow). Sagittal image (**B**) shows the relation of the cyst to the S2 foramen

Perineural cysts (Tarlov cysts) can belong to either Nabor’s group I or II, with or without spinal nerve root fibers. Anatomically, Tarlov cysts are cerebrospinal fluid (CSF)-filled sacs that commonly occur at the junction of the posterior root and the dorsal ganglion appearing as gross dilations of spinal nerve root sleeves [5]. The cyst occupies the space between the perineurium (arachnoid covering the nerve root) and the endoneurium (outer layer of the pia). Nerve root fibers, and occasionally ganglion cells, exist within the cyst wall or freely in the cyst, and the entire cyst may be surrounded by neural tissues [6, 7]. They commonly exist at the sacral level (Fig. 1) but have been reported to exist at other spinal levels including the lumbar and cervical regions [8–11].

The pathogenesis of Tarlov cysts remains unclear. Multiple hypothesis including inflammation, trauma, congenital origin, and degenerative processes have been proposed [12, 13]. Although acquired conditions of cyst formation have previously been proposed to involve inflammation or trauma [7, 14, 15], the currently accepted cause of cyst development has been cited as a disruption of the CSF-venous drainage mechanism at the perineurial-epineurium junction [6, 7, 15]. There is also a general view that cyst enlargement occurs because a microcommunication exists between the cyst and the subarachnoid space and that a ball-valve-type mechanism allows CSF influx and restricts efflux leading to an expansion of the cyst [5, 16–18].

Congenital causes have included dural ectasia referring to connective tissue weakening, ballooning or widening of the dural sac, or arachnoid proliferations within the nerve root sleeve with obstruction of normal CSF return often precursors to the development of meningeal cysts, an interchangeable term for Tarlov cysts. Usually, the sacral region is the most vulnerable area for these abnormalities due to the presence of the highest pressure of CSF. However, these effects can occur at any level of the spinal column regardless of the point of pressure exertion.

**Fig. 2:**
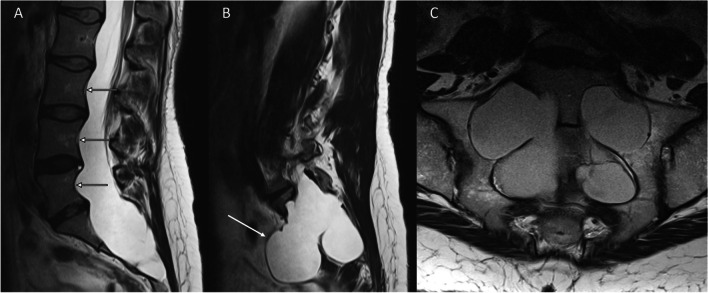
T2-weighted sagittal and axial images of the lumbosacral spine which demonstrate dural ectasia in a patient with Marfan’s syndrome. **A**, **B** Sagittal images illustrate posterior vertebral body scalloping (arrows) and anterior sacral meningoceles (arrows). **C** Axial image shows marked enlargement of the neural foramina associated with dural ectasia. Dural ectasia is a feature of other hereditary connective tissue disorders including Loeys-Dietz and Ehlers-Danlos syndrome

Dural ectasias can exist in isolation or be associated with neurofibromatosis [19, 20], ankylosing spondylitis [21, 22], or heritable connective tissue disorders such as Marfan syndrome (Fig. 2) [23–25], Ehlers-Danlos syndrome [26, 27], or Loeys-Dietz syndrome [28]. These syndromes are hereditary disorders that compromise the strength and elasticity of connective tissues throughout the body, including those of the dura. Dural ectasia is a frequent finding with a reported incidence of 63% within a 57-patient Marfan cohort with no cases present in the age-sex-matched non-Marfan control group [24]. These patients have also frequently reported diverse pain and neurological dysfunction symptoms associated with dural ectasia [29]. However, these syndromes are rare, and the true prevalence of Tarlov cysts occurring with them is unknown reported only in case reports [30–32].

## Prevalence of Tarlov cyst

The prevalence of these cysts has been greatly underestimated as they are often asymptomatic and detected incidentally on CT or MRI examinations performed for a history of back pain or radiculopathy. Prevalence of Tarlov cysts has been investigated in MRI exams performed in nine studies conducted in different countries [5, 13, 33–39]. The studies detailed in Table 1 involved retrospective reviews of imaging investigations for large numbers of adult men and women; two studies [5, 36] included children and adolescents.

**Table 1:** Incidental radiographic findings and prevalence of Tarlov cysts

Author, report year, country	Study sample	Tarlov cyst prevalence	Summary
Burdan [33] 2013 Poland	• Between January 2011 to December 2012, 842 patients (543 women, 145 men) • Underwent 1.5 T MRI in the cervical, thoracic, lumbar and sacral spine regions	• Overall Tarlov cysts 8.9% (75/842) • Sacral Tarlov cyst 7.6% (64/842)	• Cysts occurred as single cysts (29.3%) or multiple cysts (70.7%) • Tarlov cysts mainly occurred at the S2 (*n* = 22) and S2/S3 (*n* = 20) sacral levels • Cyst patterns occurred at all other spinal levels but rarely cervical cysts (*n* = 3) all occurred in women • Cyst incidence was higher in women than men,
Joo [34] 2009 South Korea	• Between January 2006 and June 2009, 2669 patients (1633 women, 1036 men) • Primary complaints low back pain or sciatica visited the outpatient Dpts of Anesthesiology, Orthopedic Surgery, Neurosurgery, Neurology and Rehabilitation Medicine • Underwent lumbosacral MRI	• Sacral Tarlov cysts 2.5% (67/2669)	• Cysts located below sacral S3 level (*n* = 21) • Lower lying terminations of dural sacs (below the S3 sacral vertebrae) were found in 1.6% of the 2669 cases • The abnormal position of the dural sac termination results in an more inferiorly extended spinal cavity and presence of Tarlov cysts in the sacrum presented an increased risk of dural puncture with caudal blocks
Kuhn [36] 2017 France	• 1000 adults (604 women, 396 men) age range 18–94 years and 100 children and adolescents (age range 2 months–17 years • Underwent 1100 consecutive sacral 1.5 T MRI	• Sacral Tarlov cysts 13.2% (132/1000)	• Cysts (*n* = 263) were found in 132 adults (101 women, 31 males), none were found in children or adolescents • Prevalence of cysts increased by age • Mean cyst size 12.8 ± 5.6 mm and mean cyst number was 2.0 ± 1.2 per patient, maximum number was 6 cysts • Cysts mainly affected the S2 (*n* = 71) and S3 (*n* = 3) sacral nerve roots • Unusual cyst architecture occurred: endocystic nerve fiber crossing (*n* = 28), septations (*n* = 5), bone erosions (*n* = 70), endopelvic extension (*n* = 13)
Langdown [37] 2005 Australia	• Over 5 years, 3535 patients • Investigations for lower back pain, sciatica or spinal stenosis • Underwent lumbosacral MRI	• Sacral Tarlov cysts 1.5% (54/3535)	• 54 sacral cysts (38 women, 16 men) • Symptoms included low back pain (*n* = 30), nerve root pain (*n* = 17), leg pain (*n* = 9), neurological loss (*n* = 5), spinal claudication (*n* = 5) with durations from months to years • Identified 3 groups based on relationship of symptoms to pathology: group 1, symptoms thought unrelated to sacral cyst (*n* = 38); group 2, sacral cyst possibly contributory but thought not to be primary cause (*n* = 9); group 3, sacral cyst directly responsible (*n* = 7)
Park [13] 2011 South Korea	• Between January 2007 and October 2009, 1268 patients (847 women, 421 men) • Presenting symptoms of chronic back pain and radiating pain • Underwent lumbar 1.5 T MRI for clinically suspected herniated intervertebral disc	• Overall incidental findings 8.4% (107/1,268) • Sacral Tarlov cysts 2.1% (27/1,268)	• Occurrence of pathologies – fibrolipoma (3.2%), Tarlov cysts (2.1%), vertebral hemangiomas (1.5%), synovial cyst (0.8%) and sacral meningocele (0.8%)
Paulsen [5] 1994 Unite States	• 500 consecutive patients (265 women, 234 men) age range 11 to 89 years • Underwent lumbosacral MRI for low back pain	• Sacral Tarlov cysts 4.6% (23/500)	• 22% (5/23) of sacral cysts were thought to have symptoms consistent with cyst location
Senoglu [38] 2012 Turkey	• Between January 2010 and 2012, 1000 patients (641 women, 358 men) • Patients referred to a neurosurgical outpatient clinic primarily for low back pain and/or sciatica • Underwent lumbar MRI • Evaluated for sacrococcygeal abnormalities and the risk of dural sac puncture during caudal epidural blocks	• Sacral Tarlov cysts 1.3% (12/1000)	• Majority of cysts (10/12) were at or below the S3 level • Dedicated sacral MRIs were not performed
Shoyab [39] 2021 Bangladesh	• Between January 2017 and December 2019, 384 patients (202 women, 182 men) • Underwent 3.0 T MRI at all spinal levels (cervical, thoracic, lumbar, sacral) for back pain	• Overall Tarlov cyst 6.5% (25/384) • Sacral Tarlov cysts 6.0% (23/384)	• Majority of cysts were in the sacral region, 1 was in the thoracic and 1 in the cervical spine levels • Prevalence of Tarlov cysts was similar for men (6.0%) and women (6.9%) • Cysts were single level cysts in majority of patients (72%) and occurred mainly (60%) at S1/S2 sacral root level • Mean cyst diameters were significantly greater in the craniocaudal (24 ± 10.25 mm) than the sagittal (9.5 ± 2.07 mm) or transverse (12 ± 2 mm) dimensions

The primary complaint for the referrals involved lower back pain, sciatica, or other spinal pathology such as herniated disc or spinal stenosis. Investigations all involved MRI exams but several only involved lumbar MRI [13, 38] rather than lumbosacral or dedicated sacral MRIs. The prevalence of Tarlov cysts ranged from 1.5 to 13.2% with the highest prevalence reported for studies employing sacral MRIs—Shoyab 6% [39], Burdan 7.6% [33], and Kuhn 13.2% [36]. Cysts commonly occurred at multiple sacral levels, increased with age, and were more common in women. Kuhn et al. [36] reported unusual architecture in many of the 263 evaluated sacral cysts—endocystic crossing of nerve fiber and internal septations (12.5%), adjacent bone erosion (26.6%), and pelvic extension (4.9%). Klepinowski et al.’s [40] meta-analysis of 22 imaging studies reported a pooled incidence rate of 4.18% (95% CI, 2.47–6.30%) for Tarlov cysts. They also reported that Tarlov cysts occurred more commonly in women than men (7.0% vs 4.1%), rarely reported in children, frequently located in the sacrum, and occurred in both single and multiple locations with a mean 11.9-mm (95% CI, 10.8–12.9 mm) cyst diameter.

The prevalence of Tarlov cysts has been reported to be higher in female populations in studies specifically targeted at populations involving women referred for gynecological or urological conditions [41–43]. Lim et al. [42] evaluated the presence of Tarlov cysts in a 242-patient cohort referred to an academic chronic pain center with pudendal neuralgia, a chronic pelvic pain syndrome. Patients were referred for pelvic MRIs, and 16% (34 women, 5 men) of them had a least one Tarlov cyst with the majority located at the S2–S3 sacral levels.

Tani et al. [43] evaluated 102 consecutive Japanese women, mean age 41.4 years (range 22–77 years) with gynecological problems who underwent pelvic MRI and subsequently an additional sacral MRI. Ten women (9.8%) were suspected of having a symptomatic sacral Tarlov cyst with seven women (6.9%) diagnosed with a high probability of symptomatic sacral Tarlov cysts.

Hulens et al. [41] evaluated the prevalence of Tarlov cysts in a 197-patient cohort (180 women, 17 men) referred to an outpatient musculoskeletal pain clinic and diagnosed with fibromyalgia (FM) or chronic fatigue syndrome (CFS). Lumbar and sacral MRIs reviewed for Tarlov cysts were seen in 39% (75 women, 2 men) of patients, more commonly with patients having FM with or without CFS (*n* = 71). The mean cyst size was 11.8 mm (range 5–30 mm) and was significantly larger in patients older than 50 years of age (12.7 ± 5.5 mm) than in those younger than 50 years of age (10.5 ± 4.6 mm).

## Clinical impact

For many years, radiologists have reported Tarlov cysts as an incidental finding of doubtful clinical significance and giving greater prominence to other co-existing spinal pathologies [44–46]. There is, however, extensive evidence that Tarlov cysts identified radiologically can be symptomatic. Little is known about the growth of these cysts, but a natural history analysis by Yang et al. [47] with 4 years of follow-up of MR-identified Tarlov cysts reported that none of the cysts spontaneously decreased in size and that 17% increased minimally in the craniocaudal direction. In that study, positional headache symptoms associated with cerebral CSF hypotension were found on logistic regression to be significant predictors of cyst growth. Cerebrospinal fluid leakage is the cause of intracranial hypotension [48]. In one study of 568 patients with spontaneous intracranial hypotension, sacral dural ectasia/Tarlov cysts were noted in 22 patients (3.9%) [49]. Ferrante et al. [50] reported that in their series of more than 200 cases of spontaneous intracranial hypotension, CSF leaks were observed with three cases of Tarlov cysts.

**Fig. 3:**
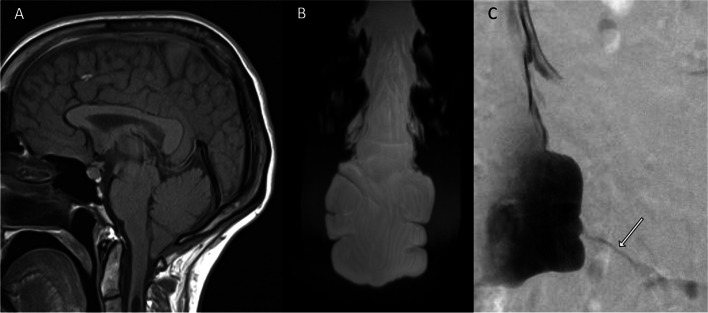
CSF-venous fistula arising from a sacral meningeal diverticulum causing intracranial hypotension. **A** T1-weighted sagittal image demonstrating features of intracranial hypotension including brainstem sagging, enlargement of the pituitary gland, dural venous sinus distension, and syringomyelia. **B** MR myelogram demonstrating a meningeal diverticulum in the sacral canal. **C** Digital subtraction myelogram showing a CSF-venous fistula (arrow). Case provided courtesy of Dr Wouter Schievink

Tarlov cysts have been associated with CSF leakage and intracranial hypotension [14, 51] although a spinal CSF leak should not be ascribed to a Tarlov cyst without confirmation of the Tarlov cyst as the leak site. Patients with Tarlov cysts often have other smaller meningeal diverticula along the spinal axis, and one of those diverticula may be the source of the CSF leak. Spinal CSF-venous fistulas are a recently described type of CSF leak that can also cause spontaneous intracranial hypotension [48, 52]. These fistulas are not visible on routine CT-myelography, and they require more specialized imaging, such as digital subtraction myelography, for their detection. Most of these fistulas are found in the thoracic spine [53], but they may also be seen arising from the sacrum (Fig. 3) [54, 55]. There are reported cases of associations of Tarlov cysts with hydrocephalus [56] and idiopathic intracranial hypertension [57].

An increasing cyst size can have an adverse impact in other ways. The increase in size may result in distorting, compressing, or stretching nerves running through the cyst or compressing neighboring spinal nerve fibers resulting in various neurological symptoms depending on the spinal level of involvement [58]. Cysts may also become large enough to erode surrounding bony tissue causing irritation of periosteal pain fibers and in rare cases have been responsible for sacral insufficiency fractures [59, 60]. Although cysts rarely become large enough to spontaneously rupture, they have been reported in case reports to rupture with CSF leakage resulting in intracranial hypotension [16, 61]. Two unusual case reports dealt with a cerebral fat embolism after a traumatic rupture of a Tarlov cyst secondary to a sacral fracture after a fall with a low back injury [62, 63]. The fatty bone marrow was believed to have migrated from the sacral fracture to the brain through a dural breech of the cyst.

Endopelvic extension of sacral cysts is uncommon, but presacral pelvic meninogoceles may become large enough to extend through the anterior sacral neural foramina into the pelvic cavity. There have been multiple case reports [12, 64–68] of patients, mainly women, initially presenting with abdominal or pelvic pain that were frequently misdiagnosed on ultrasonography, as unspecified gynecological masses. Various diagnoses have been initially proposed for these conditions such as hydrosalpinx, ovarian cyst, para-ovarian cysts, or adnexal mass. An unusual case was reported of a giant Tarlov cyst extending into the presacral space causing ureteric compression resulting in hydronephrosis in a woman with Marfan syndrome [31]. These missed diagnoses have resulted in delays, unnecessary laparoscopic or laparotomy procedures, and in some cases inappropriate treatments [65, 67].

Although Tarlov cysts have been reported in some cases to be associated with isolated lower limb radiculopathy and paresthesias [69, 70], they are also more commonly associated with pelvic, perineurial, urogenital pain, and neurological conditions. The broader impact of Tarlov cysts was detailed more extensively in clinical assessments of six cohorts with MRI-identified sacral Tarlov cysts involving different referral patient groups [37, 42, 71–74]. The assessments and symptom profiles of patients with Tarlov cysts in these studies are detailed in Table 2.

**Table 2:** Symptoms associated with Tarlov cysts

Author, year, country	Referral cohort, investigations	Tarlov cyst details	Presenting symptoms
Langdown [37] 2005 Australia	• 54-patient cohort (38 women, 16 men) mean age 54.4 years (range 27–83 years) with symptom duration months to years • Referred over a 5-year span for a spinal surgeon specialist opinion for low back pain, sciatica or spinal stenosis	• All cysts occurred in the sacral region • Cysts were frequently multiple in various locations, unilateral, central or bilateral	• Low back pain (*n* = 30), nerve root pain (*n* = 17), leg pain (*n* = 9), and neurological loss (*n* = 5)
Marino [73] 2013 Italy	• 157-patient cohort (138 women, 19 men) mean age 48 ± 11 years with a mean age at symptom onset at 42 years of age • Between October 2008 and May 2012 referred to a neurosurgical outpatient clinic • Underwent neurological investigations (complete electroneurography of lower limbs), bowel/bladder and gynecological exams • Pain scored by VAS and depression by the Hamilton Depression Rating Scale	• Cases involved single or bilateral sacral cysts • Lumbar cysts (*n* = 3)	• Almost all with perineal or lower back pain • MRI evidence lumbar and/or sacral disc herniation (95%) • Sphincter disorders (34.4%) similar for women and men) • Sexual dysfunctions higher for men vs women (36.8% vs 28.2%) • Electroneurographic findings (*n* = 32) were normal • Pain mean VAS score 4.7 ± 2.0 and depression rating scale 18.1 ± 10.4 in the mild severity range (similar for men and women) • Social psychological effects included mood disorders, social, employment issues and loss of jobs
Murphy [74] 2016 Canada	• 213-patient cohort, 289 cysts • Between 2003 and 2012 referred to an interventional neuroradiologist and spine neurosurgeon • Underwent lumbar and dedicated sacral MRI, physical exam, neurological assessment and/or gynecologist or urologist evaluation if pelvic, abdominal or genital symptoms were present • Pain and function assessed by Lumbar Spine Outcomes Questionnaire	• Cysts involved single nerve roots unilaterally (*n* = 113), single roots bilaterally (*n* = 78) and more than two roots bilaterally (*n* = 22) • Location mainly S2 (*n* = 142) and S3 (*n* = 120) sacral levels • Cyst size ranged between 2 and 4 cm (*n* = 143), 8 cysts were greater than 4 cm	• Generalized sacral and/or lumbar pain (89%) • Pelvic pain and/or perineal pain (98%) • Bladder (43%), bowel (29%) • Sexual dysfunctions (43%) • Common neurologic abnormalities included absent Achilles reflex (61%), plantar flexion weakness (41%), reduced rectal sphincter tone (29%), bladder sphincter impairment (43%), cyst-related sensory loss at S1 (8%), S2 (64%), S3, S4, S5 (46%)
Baker [71] 2018 United States	• 65-women cohort, mean age 53 ± 12 years, 89% White • Between July 2004 and October 2015 referred to urogynecology or neurosurgery clinics • Indication for clinic visit, back pain, radicular pain, pelvic pain, sexual dysfunction urinary or bowel dysfunction • Medical and surgical history, physical exam detection of lower extremity numbness or weakness and urodynamic testing	• Sacral cysts mainly located at the S2 or S2/S3 sacral regions • Cyst mean size 17 mm (range 5– 59 mm) • Sacral cysts larger than 10 mm (90%)	• Symptoms by region: lumbosacral (95%), urinary (78%), bowel (55%), central nervous system (40%) and sexual dysfunction (25%) • Commonly reported low back pain (83%), lower extremity pain (75%), positional pain arising to sitting or standing (62%), urinary urgency (54%) and urinary frequency ((48%) • Urodynamic testing (*n* = 40) with 78% having abnormalities with early bladder filling sensation the most frequent finding • Urinary urge incontinence (31%) vs 7% general population
Hulens [72] 2018 United States	• 33-patient cohort (30 women, 3 men) mean age 49.8 years • Study data collected July 2015 and October 2016 • Comparator group, 42 age-sex matched patients (38 women, 4 men) experiencing long-term back pain or sciatica due to degenerative disorders or inflammatory disease • Nerve conduction tests and electromyography for lumbar and sacral nerve root myotomes • Symptom profiles based on modified International Tarlov Cyst Questionnaire (Feigenbaum 2022)	• Study restricted to Tarlov cysts ≥ 15 mm in diameter	• Symptomatic for mean 9.5 years and were significantly more common in patients with Tarlov cysts vs those with only back pain • Lower limbs [foot pain (24% vs 10%), leg pain (25% vs 5%), foot paresthesia (31% vs 5%), and subjective leg weakness (72% vs 28%)] • Pelvic symptoms [perineal paresthesia (24% vs 5%) dyspareunia (32% vs 5%), and coccygodynia (49% vs 17%)] • Bowel and sphincter [constipation (63% vs 33%), anal pain (57% vs 10%), mild fecal incontinence (55% vs 7%)] • Bladder and urinary sphincter: hesitation (38% vs 5%), retention (55% vs 17%), and frequency (69% vs 38%) • Pain aggravating features sitting (100% vs 74%) or long walks (75% vs 34%) • Reduced social functioning (78% vs 18%), forced to stop working (54% vs 18%)
Lim [42] 2020 United States	• 39-patient cohort (34 women, 5 men) mean age 51.3 years (range 24–83 years) • Between January 2010 and November 2012 referred to an academic chronic pain center having MRI-identified Tarlov cysts and pudendal neuralgia (validated by Nantes criteria), a chronic pelvic pain syndrome	• Cysts mainly located at S2–S3 sacral level (64%) • One cyst outside the pudendal nerve distribution (L5-S1) • Cyst sizes 44% small (< 1 cm), 50% moderate (1–2 cm)	• Pain locations for women in the perineum (*n* = 7), rectum (*n* = 14), and vagina (*n* = 19) and for men in the perineum (*n* = 4) penis (glans) (*n* = 2) and scrotum (*n* = 3) • Comorbid conditions for women including interstitial cystitis (*n* = 4), pelvic floor tension myalgia (*n* = 17) and pelvic organ prolapse (*n* = 7), persistent genial arousal disorder (PGAD) (*n* = 2) • Pelvic floor tension myalgia in men (*n* = 2)
Cattaneo [83] 2001 Italy	• 11-patient cohort referred to an outpatient EMG laboratory with lumbosacral MRI documented sacral Tarlov cysts • Sural nerve investigated electrophysiologically	• In 5 patients with sural nerve abnormalities, cysts size ranged from 2 to 3 cm, were located bilaterally (*n* = 3), or unilateral right (*n* = 2) and at the S1 to S3 sacral levels	• Symptoms included sciatica (*n* = 3), bilateral foot paresthesia and pain (*n* = 1) and 1 was asymptomatic • Sural sensory abnormalities found in five patients (2 women, 3 men) aged 40 to 66 years • Abnormalities were localized to the side of the Tarlov cysts and the size of the cyst was not related to the presence and extent of the nerve damage • Motor nerve conduction was spared demonstrated with normal findings in all subjects accounted for by the location of the cysts at the dorsal root
Hulens [84] 2016 Belgium	• 3 case reports (3 women) • Sural nerve conduction studies and electromyelographic tests evaluating nerve injury and relating to patients’ symptoms	• Multiple Tarlov cysts ranging in size from 6 to 36 mm	• Symptoms included radicular back leg pain, urinary/fecal sphincter disturbances, perineal pain, dyspareunia, and headaches • EMG findings varied in patients with delayed sural nerve response and ano-anal reflex occurring
Hulens [85] 2017 Belgium	• 30-patient cohort (27 women, 3 men) with Tarlov cysts mean age 46 years (range 25–74 years) • Referred to a physical medicine outpatient clinic for unexplained refractory low back, pelvic, perineal and/or leg pain, symptomatic for mean 11.6 ± 12.0 years (range 8 months and 50 years) • Needle EMG studies to evaluate lumbosacral dermatomes	• Cysts located on L5 to S4 nerve roots were commonly bilateral with a mean size of 7.1 mm (range 3 to 36 mm) • Nerve root dilations or smaller Tarlov cysts (< 10 mm) were found in the cervical (C7) to thoracic (TH4) regions for most (23/30) patients	• Symptoms included pain in lower back/sacral pain (83.3%), buttock (80%), coccygeal 47%, perineal 33%, dyspareunia/genital pain (69%); parasthesis in perineum (47%), buttocks (47%), legs (63%), foot/feet (80%) – foot drop (3.3%); bladder dysfunction (77%), bowel dysfunction (70%) • Increased pain with sitting (96.7%) or standing (86.7%) • Sural nerve conduction abnormalities (16.7%) with 4 of the 5 dermatomal abnormalities corresponded to dermatomal pain and/or paresthesia • S1 Hoffman-reflex latency abnormality (23.3%) with 7/7 corresponding to dermatomal pain and/or paresthesia • Ano-anal reflex abnormality (89.3%) with 23/25 corresponding to dermatomal pain and/or paresthesia • Needle EMG abnormalities were found at all levels: L4 (36.7%), L5 (76.7%), S1 (40%), S2 (96.6%), and S3–S4 (75%), with abnormalities corresponding to Tarlov cysts on that nerve root: L4 (7/18), L5 (33/49), S1 (25/58), S2 (32/56), and S3-S4 (37/59)
Hulens [86] 2022 Belgium	• 31 patients (26 women, 5 men) mean age 47 ± 10 years (range 31–74 years) with symptomatic Tarlov cysts larger than 8 mm • Lumbar and sacral nerve root abnormalities investigated for large- and small-fiber neuropathy • Investigations included EDX tests (*n* = 24) included nerve conduction and needle EMG, and lower leg skin biopsies to assess IENFD (*n* = 17), 11 patients underwent both IENFD and EDX	• Cysts located mainly in the sacral areas varied in mean size: L4 (*n* = 3), 8.7 ± 1.2 mm (range 8–10 mm) L5 (*n* = 11), 8.5 ± 0.8 mm (range 8–10 mm) S1 (*n* = 24), 10.8 ± 2.6 mm (range 8–17 mm) S2 (*n* = 28), 12.6 ± 6.2 mm (range 8–34 mm) S3 (*n* = 23) 14.5 ± 6.1 mm (range 8–35 mm), S4(*n* = 6), 14.3 ± 3.4 mm (range 10–20 mm)	• IENFD was < 5th percentile for age-sex in 82% • Nerve conduction abnormalities included delayed sural nerve response latency (6%, 1/16), low sural nerve amplitude (13%, 2/16) and delayed Hoffman reflexes (25%, 5/20) • Anal reflexes were delayed in 95% (20/21) and 57% (12/21) patients with delayed anal reflex latency reported mild (48%) to severe (10%) fecal incontinence • EMG during voluntary contraction showed patchy neurogenic motor unit potentials of mild severity mainly at the L5 (72%, 13/18), (82%, 18/22) and S3–S4 (82%, 18/22) myotomes • EMG abnormalities of myotomes generally did not correspond to cyst location

*EDX* electrodiagnostic studies, *IENFD* intraepidermal nerve fiber density

Langdown et al.’s [37] early report of 54 Australian patients (38 women, 16 men) with MRI-identified sacral Tarlov cysts were referred for a spinal surgeon specialist opinion for low back pain, sciatica, or spinal stenosis. Their objective was to clarify the relationship of Tarlov cysts in the origin of symptoms of lumbosacral spinal canal stenosis focusing only on typical symptoms of low back pain, nerve root pain, leg pain, and neurological loss. Based on these restricted symptoms, the authors concluded that only 30% of patients had symptomatic Tarlov cysts that could be considered contributory, or the main cause of symptoms.

When comorbid spinal conditions are present along with Tarlov cysts, it can be difficult to attribute causality of symptoms. Complicating treatment decisions are observations that spinal degenerative changes themselves are known to increase with age and degenerative changes have commonly been reported in various imaging investigations in asymptomatic subjects [75–80].

This early study by Langdown et al. [37] did not investigate the occurrence of other symptoms in patients with Tarlov cysts. A focus of MRI mainly on the lumbar region and the usual degenerative spinal pathologies and absence of gynecological or urological investigations for symptoms related to sacral cysts have been cited as major reasons why the clinical impacts of sacral Tarlov cysts are often overlooked [45].

Five more recent studies investigating symptoms of patients with Tarlov cysts have involved either more extensive evaluations, different referral settings, or study groups involving mainly women [42, 71–74]. Additional investigations in these studies have included bladder, urinary, and sexual functions as Tarlov cysts located in the sacral regions can adversely impact nerves for these functions (Table 2). Enquiry into these symptoms is rare as many spine surgeons focus on the disc space and are uncomfortable discussing genital pain and sexual dysfunction [81].

Marino et al.’s [73] 157-patient (138 women, 19 men) Italian cohort with Tarlov cysts were referred to a neurosurgical outpatient clinic (Table 2). Patients were on average 48 years old with an average age of symptom onset at 42 years of age—a 6-year duration. Investigations included neurological, gynecological, and urological and although almost all reported perineal or lower back pain; sphincter disorders and sexual dysfunctions were also reported (Table 2). Social and psychological impacts were also reported with some having social and employment issues involving job loss.

Murphy et al.’s [74] 213-patient American cohort with symptomatic sacral Tarlov cysts had been referred to a spine neurosurgeon and an interventional neuroradiologist and followed up for 5 to 10 years. Patients underwent physical examinations and neurological examinations, and those with pelvic, abdominal, or genital symptoms were seen by a gynecologist and/or urologist. Most patients had generalized sacral and/or lumbar pain; pelvic pain and sexual dysfunctions were also frequently reported (Table 2). Typically, many of these patients had difficulty sitting. Multiple neurologic abnormalities were also commonly found, and two patients with cysts compressing the L5 nerve root had dorsiflexion weakness and complete foot drop.

Baker et al.’s [71] 65-women American cohort with Tarlov cysts had been referred to urogynecology or neurosurgery clinics. Patients reported one or more symptoms in diverse areas—lumbosacral, urinary, bowel, central nervous system, and sexual dysfunction (Table 2). The most frequently reported symptoms in addition to low back pain were lower extremity pain, positional pain arising to sitting or standing, urinary urgency, and urinary frequency. Many undergoing urodynamic testing had abnormalities with early bladder filling sensation the most frequent finding; the 31% reporting urinary urge incontinence was significantly more prevalent than the 1–7% prevalence cited for the general population.

Hulens et al. [72] investigated symptom profiles of 33 patients (33 women, 3 men) with Tarlov cysts symptomatic for over 9 years based on responses to a modified International Tarlov Cyst Questionnaire [82]. This study was the only one to compare responses with a comparator group, 42 age-sex-matched patients experiencing long-term back pain or sciatica due to degenerative disorders or inflammatory disease. Significantly, more symptoms were found for patients with Tarlov cysts than comparator patients in several regions including the lower limbs, pelvis, coccygodynia, bowel, and bladder (Table 2). Pain aggravating feature such as sitting or walking were also common features in women with Tarlov cysts and other impacts on their quality of life were that they were significantly more likely to reduce their social activities, stop working, and experience social decline.

Lim et al. [42] evaluated thirty-nine patients (34 women, 5 men) referred to an academic chronic pain center with pudendal neuralgia, a chronic pelvic pain syndrome, and having MRI-identified Tarlov cysts. Pain locations were reported by women in the perineum, rectum, and vagina and by men in the perineum, penis (glans), and scrotum. Comorbid conditions also existed for women including diagnoses of interstitial cystitis, pelvic floor tension myalgia, pelvic organ prolapse, and persistent genital arousal disorder (PGAD). Pelvic floor tension myalgia was also identified in two men. No significant associations were found between cyst size, pain laterality, and concordance of pain symptoms with cyst location.

### Neurophysiological studies

Several investigators [83–86] employed electrodiagnostic studies, both nerve conduction and needle electromyography (EMG) to assess axonal or nerve root injury associated with Tarlov cysts and correlate with sacral nerve root damage (Table 2). The sural nerve was often the target nerve in the studies as it is composed mainly of fibers originating mainly from the S1 and S2 sacral roots where Tarlov cysts tend to localize. Tarlov cysts are located at the dorsal roots and primarily affect sensory functions. However, if cysts protrude or impact on the ventral region, motor conduction abilities could also be affected. Motor neuron symptoms, i.e., foot-drop, have been reported for patients with Tarlov cysts in two studies, although for a minority of patients: 2/213 [74] and 1/30 [85].

Sural nerve abnormalities were initially documented in two studies [83, 84]. In an initial small study [84], sural conduction abnormalities were reported for three patients and Tarlov cysts as small as 6 mm were found to cause nerve damage and debilitating symptoms correlating with patients’ symptoms. In another study [83], abnormalities noted in five of the eleven patients with Tarlov cysts were localized to the side of the Tarlov cysts and the size of the cyst was not related to the presence and extent of the nerve damage.

Abnormal neurophysiological findings were also reported in larger cohort studies of patients referred to a physical medicine outpatient clinic: a 30-patient cohort [85] and a later 31-patient cohort [86]. Nerve conduction studies in the 30-patient cohort were abnormal for sural nerves, S1 Hoffman-reflex latency, and the ano-anal reflex with almost all abnormalities corresponding with dermatomal pain or paresthesia [76, 85]. The S3–S4 ano-anal reflux prevents fecal incontinence, and bowel dysfunction and fecal incontinence were reported by 70% and 40%, respectively. Needle EMG abnormalities in nerve root myotomes corresponded to dermatomal pain and paresthesia with the highest correspondence at the S1/S2 and S3/S4 levels. MRIs of the cervicothoracic regions were also reviewed to rule out Tarlov cysts as a potential cause of frequent arm/neck pain complaints. Nerve root dilations or smaller Tarlov cysts (< 10 mm) were found in the cervical (C7) to thoracic (TH4) regions for most (23/30) of the patients.

Nerve fiber neuropathy was evaluated in 31 patients with symptomatic Tarlov cysts [86]. Small fiber neuropathy was evaluated with lower leg skin biopsies to assess intraepidermal nerve fiber density, and the majority of patients with biopsy samples were below the 5% percentile of age-sex-matched reference values for this nerve damage. Small fibers are responsible for nociceptive processing, thermal sensation, and autonomic functions, and damage to these fibers may produce commonly reported Tarlov cyst-related sensory symptoms such as burning pain, hyperesthesia, and dysesthesia. Nerve conduction studies were again abnormal for the ano-anal reflex and were seen as probable cause of the mild to severe fecal incontinence in over half of those reporting these symptoms. Needle EMG abnormalities were also found in the lumbar and sacral nerve roots in all patients.

Based on their findings, the authors proposed that the increased pulsatile CSF pressure that initiates Tarlov cyst formation might also be damaging axons and neurons inside the nerve root sheaths and dorsal root ganglia. The authors also noted that these abnormalities are often missed, as in practice needle EMGs are more commonly performed for diagnosis of radiculopathies related to disk herniations and only myotomes L3 to S1 are generally examined but not the sacral roots S2 to S4 where Tarlov cysts commonly locate.

## Screening

Although many Tarlov cysts are asymptomatic [37, 74, 87], the remainder can present with a range of various neurological, musculoskeletal, urological, and/or gynecological symptoms which could be attributed to numerous pathological processes. Therefore, before any intervention, it would also be important to determine the relationship between the cyst and presenting symptoms—directly related to the symptoms, an additive factor in the presence or other comorbid pathologies, or unrelated to symptoms.

Patient selection is key, and initial assessments should include a careful history and physical examination before any treatment decisions. However, patients with Tarlov cysts often present with diverse symptoms unrelated to their cysts requiring extensive intake assessments and dedicated staff to assist with screening can greatly facilitate identifying patients with symptomatic Tarlov cysts [74]. Screening has also become more difficult with patients becoming aware of their Tarlov cysts and self-referring for treatment after internet-based searches resulting in increasing numbers of ineligible patients.

**Fig. 4:**
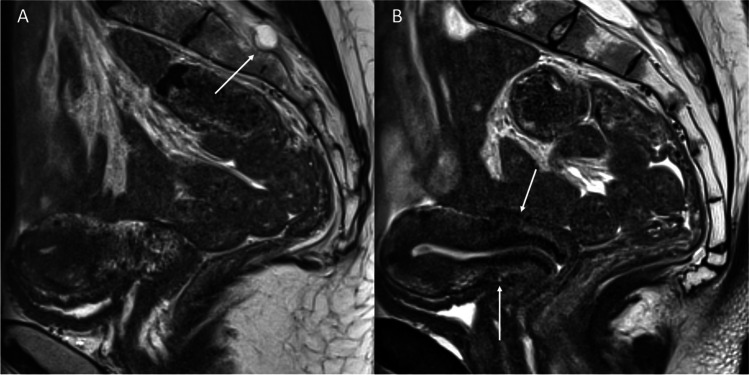
T2-weighted MR images of the pelvis in a 29-year-old female with a Tarlov cyst referred for assessment of pelvic pain. A dedicated pelvic MR was obtained in view of a history of cyclical pain. **A** Sagittal image shows the Tarlov cyst associated with the right S3 nerve. **B** Sagittal aligned-with-cervix image shows T2-hypointense plaques along the dorsal uterine serosal surface and vesicouterine recess (arrows) which are consistent with endometriosis

**Fig. 5:**
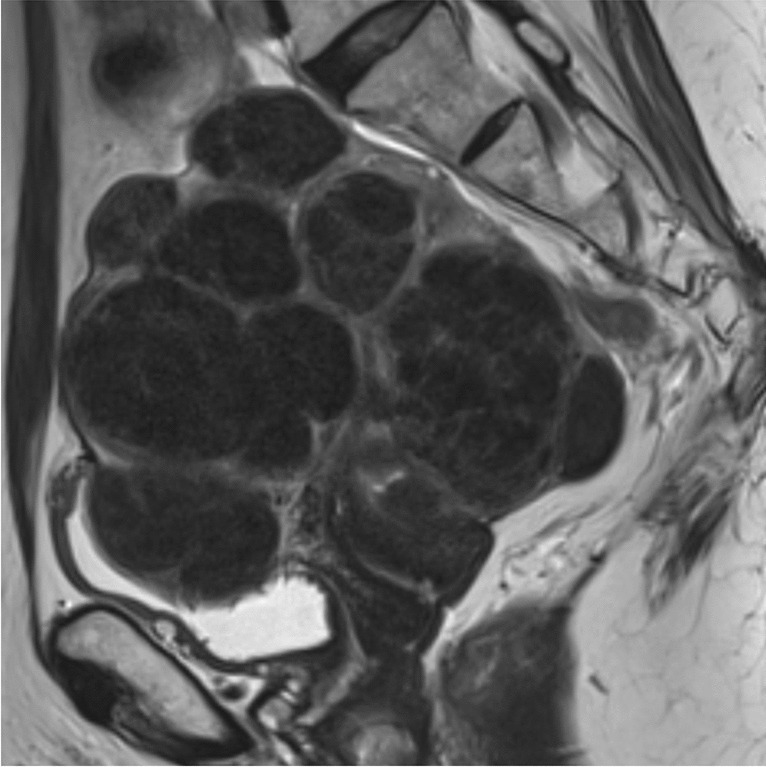
T2-weighted sagittal image of the pelvis demonstrating multiple large T2 hypointense fibroids which occupy most of the pelvic cavity and are associated with significant mass effect on surrounding pelvic structures

**Fig. 6:**
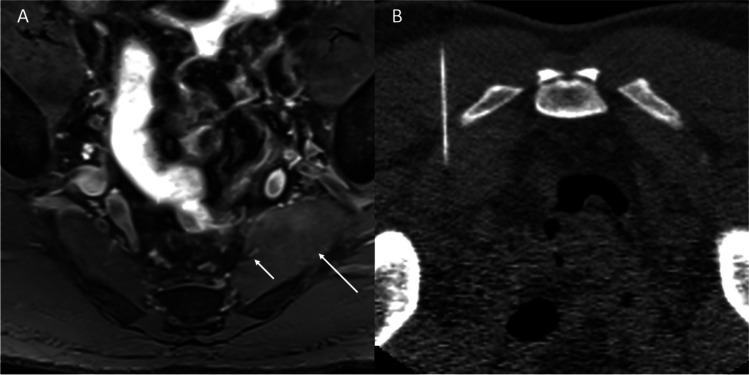
T2 SPAIR axial image (**A**) of the pelvis showing hypertrophy of the left piriformis muscle (arrow) in a male patient with left gluteal pain. The left S2 nerve (short arrow) has an intramuscular course within the medial edge of the left piriformis muscle. A CT-guided botox injection (**B**) of the enlarged left piriformis muscle was subsequently performed and provided satisfactory relief of symptoms

**Fig. 7:**
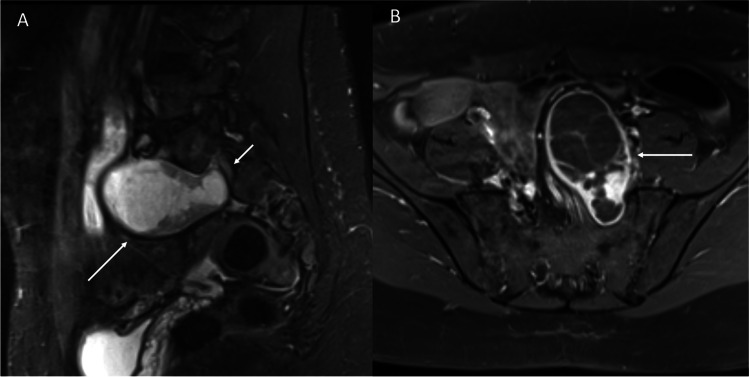
Left S1 schwannoma in a 41-year-old female who presented with chronic left-sided pudendal neuralgia. Sagittal STIR (**A**) and contrast-enhanced T1 fat-saturated axial images (**B**) show a well-circumscribed heterogenous cystic mass (arrows) arising from the left S1 nerve (short arrow) which remodels its respective foramen and extends into the pelvis

Many extraspinal origins of back or radicular pain need to be considered [88] particularly for women commonly presenting with Tarlov cysts. Women presenting with pelvic, abdominal, or genital symptoms in addition to back pain suggest consultations for further assessments by a gynecologist or urogynecologist. A range of potential gynecologic conditions including endometriosis (Fig. 4), uterine fibroids (Fig. 5), piriformis syndrome (Fig. 6), or compression from gynecologic masses (Fig. 7) could be causative or contributory to symptoms and must be excluded before treating the cysts.

Patient-completed dermatome maps have also been helpful to identify patients with Tarlov cyst-related symptoms [72]. Mapping evaluates symptoms relationship to the sacral anatomic location of the cyst and that symptoms are in the appropriate distribution of the cyst-bearing nerve roots. Electrodiagnostic assessments involving needle EMG tests and nerve conduction studies, particularly in the sacral nerve roots to assess potential nerve root injury as discussed earlier, may also provide valuable information, particularly for patients presenting with severe neurological symptoms but no abnormalities on physical exam.

The use of a modified version of the International Tarlov Cyst Questionnaire has been shown to be helpful in discriminating between symptom profiles of patients presenting with Tarlov cysts from those presenting with back pain and sciatica due to other common spinal pathologies [72]. The questionnaire has been subsequently validated as the Tarlov Cyst Quality of Life Scale, an 11-item questionnaire [82]. As discussed earlier, a range of symptoms particularly bowel and bladder dysfunctions were commonly reported by patients with Tarlov cysts. Pain associated with sitting was particularly indicative of sacral pain.

## Diagnosis

Several diagnostic modalities are available to identify Tarlov cysts. A comprehensive imaging panel should include a lumbar MRI and a dedicated sacral MRI as it is the preferred modality to detect sacral Tarlov cysts since it is more sensitive than CT scans, pelvic or routine lumbosacral MRIs [64, 74, 89]. Sacral MRI evaluation in axial and sagittal planes and should be performed with attention to the field of view, matrix, slice thickness, and positioning for anatomic localization. It is important to differentiate the neck of the Tarlov cyst as narrow- or wide-necked with a high flow of CSF as communication with the subarachnoid space can increase risk with any interventions in the sacral area [74]**.** A wide-necked cyst would be a contraindication to percutaneous fibrin sealant injections and can be distinguished by MRI-based signal in the cyst. In wide-necked cysts, connections may be visible on T2-weighted sequences, and in narrow-neck cysts, the T2 signal in the cyst will be higher than the signal in the adjacent intrathecal subarachnoid space. If MRI fails to define this connectivity, further evaluation with myelography may be needed to look for rapid contrast filling of the cyst indicating a wide connection to subarachnoid space. CT is also useful to evaluate any bone erosion by the cysts, and axial and sagittal CT is very useful to evaluate bone remodeling and plan interventions.

**Fig. 8:**
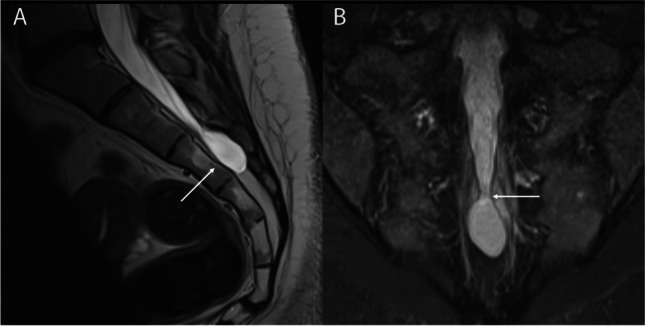
Sacral meningeal diverticulum in a 33-year-old patient. **A** T2-weighted sagittal image of the sacrum shows the meningeal diverticulum enlarging the sacral canal at the S3 level (arrow). **B** Coronal STIR demonstrates the wide-neck of the diverticulum (arrow) across which CSF is in free communication with the subarachnoid space

**Fig. 9:**
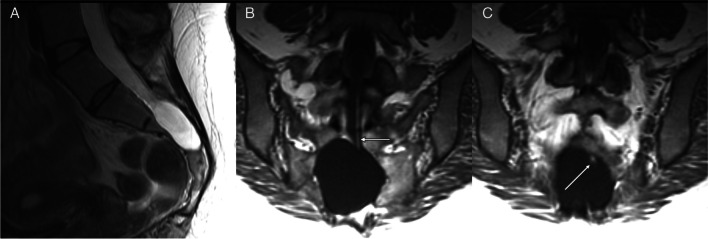
**A** T2-weighted sagittal image of a meningeal diverticulum remodelling the sacral canal. **B** T1-weighted coronal image of the sacrum demonstrating a subtle lipoma of the filum terminal (arrow) associated with the meningeal diverticulum. **C** The fatty filum traverses the neck and merges before merging with the wall of the diverticulum (not shown)

If a cyst is identified, it is very important to distinguish it from other similar conditions such as dural ectasia, meningeal diverticula (Fig. 8), or lipoma of the filum terminale (Fig. 9) which are congenital and rarely operated on [13]. Once the diagnosis of Tarlov cyst has been established and other potential pain causes have been ruled out, it is important to confirm that pain is in the immediate anatomic region of the cyst and that radicular signs and symptoms are in the appropriate distribution of cyst-bearing nerve roots, with accurate dermatomal charting. If symptoms are subjectively uncertain, the next recommended step can be to perform a diagnostic test either with local anesthetic nerve root block or by aspirating cyst fluid [81]. If pain is objectively improved after diagnostic procedures, it is likely that presenting symptoms are attributable to the cyst and further interventions could be warranted.

## Treatments

Treatment options for symptomatic Tarlov cysts range from conservative medical approaches including analgesic, anti-inflammatory, or neuropathic medications and physical therapy; minimally invasive image-guided percutaneous interventions; and various open and microsurgical approaches [44]. Those with symptomatic Tarlov cysts not responding or failing conservative medical management strategies and following confirmatory investigations that cysts are symptomatic can be considered for more invasive treatment interventions.

### Percutaneous minimally invasive interventions

Several investigators have reported case reports of successful epidural steroid injections relieving symptoms associated with sacral [90], lumbar, and cervical Tarlov cysts [10, 91]. Although injections relieved symptoms for a short term and in some cases decreased cyst size, repeated injections over time were needed to sustain treatment effectiveness. These injections, however, could also serve as a diagnostic confirmation that Tarlov cysts are the likely source of symptoms and would be helpful to indicate whether a patient would be eligible for further interventions targeting the sacral cysts [73].

Minimally invasive percutaneous approaches have also involved image-guided aspiration of CSF and injection of fibrin sealant. Fibrin sealants have been commercially available since 1998 and have been extensively used in various surgeries for its hemostatic and adhesive properties [92]. Initially, only cyst aspiration of CSF was performed and treatment success was reported in several case reports of sacral Tarlov cysts [5, 93, 94]. However, pain relief was generally short-lived, and multiple serial aspirations were often required to maintain symptom resolution.

Since then, injection of fibrin sealant following fluid aspiration has been reported by several investigators for symptomatic sacral Tarlov cysts [44, 46, 74, 95–97] and symptomatic sacral arachnoid cysts [98]. Treatment results of the cohort studies, none involving randomization, are summarized in Table 3. In all studies, patients presented with multiple symptoms that in some reports had been present for years [95, 96]. Although treatment success was defined differently in these studies, all reported high rates of symptom improvement greater than 70% after aspiration-fibrin sealant injection. Follow-up with MRI imaging also demonstrated that cysts either disappeared or were substantially reduced in size in many cases [95, 96].

**Table 3:** Treatment outcomes for cyst aspiration and fibrin sealant injection of symptomatic Tarlov cysts

Author, year, country	Study cohort, investigations	Treatment outcomes	Complications, failures
Patel [97] 1997 United States	• Consecutive series 4 patients (3 women, 1 man) with MRI documented sacral cysts, age range 40 to 62 years • Symptomatic for years with severe low back pain (*n* = 3), back pain and urinary incontinence and urgency (*n* = 1), severe perineal pain with urinary difficulty (*n* = 1) • All underwent initial cyst aspiration later followed by fibrin sealant injection on first recurrence	• Treatment successful in all cases within days of the procedure	• No symptom recurrence in follow-up from 7 to 23 months • Presumed cases of aseptic meningitis occurred with low-grade fever, headache, nausea, vomiting, meningismus (neck stiffness) and a lumbar puncture with negative cultures (*n* = 3)
Hiers [44] 2010 United States	• 130-patient consecutive cohort, retrospective review • Symptomatic Tarlov cysts treated between 2005 and 2010 • Cysts NR • Outcomes assessed by third-party review coupled with medical record reviews	• Treatment success of 75% based on several criteria: improving signs and symptoms, not wanting further treatment, and willingness to undergo another procedure if needed	• Initial 25% failure rate, a 5% failure rate occurred over time and patients not improving within 3 months; did not improve later • No post-operative infections or nerve injuries • Complications (*n* = 3, 3.8%) • Cutaneous allergic reaction (*n* = 1) • Substantial worsening of pain lasting 2 weeks then subsiding (*n* = 4)
Murphy [46] 2011 United States	• 122-patient cohort (102 women, 20 men), mean and median age 54 years • 100 patients treated between April 2004 and November 2007 for symptomatic MRI confirmed Tarlov cysts, 6 were not candidates for aspiration and refused surgery • The majority experienced lower back and buttock pain and sacral dermatome radicular symptoms with pain and burning in the perineal region, buttocks and lower extremities with 9 patients reporting bowel dysfunction • 34% presented with confounding conditions	• Improvement in symptoms (65%) and marked/total improvement (19%) • Those ineligible for cyst aspiration underwent surgical repair (*n* = 28), 63% improved symptoms	• No postprocedural fevers, or aseptic meningitis • Complication (*n* = 8, 6.6%) • Transient postprocedural sciatica (*n* = 6), rectal fullness (*n* = 1) •Postoperative urticarial resolving during overnight admission and discharged the following morning in good health (*n* = 1)
Jiang [96] 2015 China	• 42-patient cohort (22 women, 20 men) mean age 34.3 years (range, 22–56 years); mean disease duration, 20.8 months; range, 7–59 months • Treated between June 2009 and August 2012 for symptomatic MRI confirmed sacral Tarlov cysts, evaluated in mean 24-month follow-up • Majority (*n* = 31) had a solitary cyst located at L5-S1 (*n* = 5), S1-S2 (*n* = 21), and S2-S3 (*n* = 17) • Outcomes assessed pain index (VAS), functional improvement and imaging findings	• ^+^Recovery was rated as excellent (59.5%), good (26.2%), fair recovery (7.1%) and poor recovery (7.1%)— overall excellent/good recovery rating (85.7%) • Post-operatively the majority (85%) of patients had either no pain (*n* = 25) or mild (*n* = 11) pain as VAS scores (1–3) • During MRI imaging follow-up, cysts either disappeared (*n* = 25) or significantly decreased in size (*n* = 14) and did not increase in follow-up— 3 cysts no change in size during the follow-up	• Failure rate (fair or poor recovery) 14.2% • No symptom or cyst recurrences • No postoperative infection, nerve damage, meningitis or CSF leaks • Sanguineous fluid was aspirated during the procedure (*n* = 6) but no adverse effects noted • Complications (*n* = 7, 16.7%) • Headache, low-grade fever, nausea or vomiting without neck stiffness (*n* = 7) resolving with 2 day treatment of 20% mannitol 250 ml and dexamethasone 10 mg and one day of prophylactic antibiotics in addition to an average of 3 days (range, 2–4 days) of bed rest
Murphy [74] 2016 United States	• 213-patient cohort with symptomatic MRI-confirmed Tarlov cysts (*n* = 289) • Treated between 2003 and 2012 with 90.1% followed for 1 year and 83.1% followed for between 3 and 6 years • Single nerve roots unilaterally (*n* = 113), single roots bilaterally (*n* = 78) and more than two roots bilaterally (*n* = 22) • Cyst locations at S2 (*n* = 142) and S3 (*n* = 120) sacral levels, with cyst size (2 to 4 cm) • Outcomes included pain and function assessed by Lumbar Spine Outcomes Questionnaire	• ^++^Treatment overall outcomes were rated as excellent (54.2%) or good/satisfactory (27.6%) and 81% were satisfied at one year with treatment outcomes • Improvements rated as excellent or good for individual presenting symptoms: local pain (75.7%), sciatica/neuropathy (74.8%), perineal pain/sensory loss (74.9%), bladder/sexual function (73.9%), bowel dysfunction (72.6%), plantar flexion weakness (73.6%) or paralysis (0%), dorsiflexion paralysis (0%), and rectal sphincter reduction (73.8%)	• Treatment failures (18.2%) • Symptom recurrence (16.9%) • No documented infections, nerve injuries or aseptic meningitis • Complications (*n* = 44, 20.7%) • Mild nonspecific allergic reaction with systemic hives leading to overnight hospitalization resolving without incident (*n* = 1) • Elevated inflammation resolving without treatment (*n* = 3) • Spinal fluid leak requiring a blood patch for control (*n* = 7) • Increased sciatica post-operatively resolving within 3 months (*n* = 20) and increased sciatica persisting for 3 months eventually resolving (*n* = 1) • Severely increased local pain (*n* = 7) resolving (*n* = 6) within 3 months • Increase in symptoms, including bowel and bladder dysfunction, immediately following injection, transient and resolving within 3 months (*n* = 3)
Jiang [95] 2017 China	• 82-patient cohort (49 women, 33 men) mean age 45.2 years (range 19–74 years), mean symptom duration 35.4 months (range, 6– 360 months) • Treated between June 2003 and August 2015 for symptomatic MRI-confirmed Tarlov cysts • Three treatment approaches over time: aspiration and fibrin sealant injection performed after 2009 (*n* = 56), open surgery performed before 2009 (*n* = 14) involving sacral laminectomy microsurgical partial cyst wall fenestration and imbrication followed by 2-day lumbar drainage, and those refusing surgical management were treated with conservative involving physical therapy, anti-inflammatory and neurotropic drugs (*n* = 12) • Cyst locations at L5-S1 (*n* = 21), S1-S2 (*n* = 46), S2-S3 (*n* = 17) • Outcomes included pain VAS scores, symptom and neurological deficit resolution	Aspiration and fibrin sealant injection after 2009 (*n* = 56) • Significant reductions in baseline mean pain NRS scores with low postoperative mean pain NRS scores 1.3 ± 1.1 • All symptoms and neurological deficits had been either completely or substantially resolved immediately after operation or during follow-up visits • MRI examinations in most patients showed that the cysts disappeared or decreased in size during follow-up visits	• No postoperative infections, nerve damage, or CSF leaks • No recurrences occurred • Complications (*n* = 7, 8.5%) • Low-grade fever, nausea, and vomiting without neck stiffness (*n* = 7) resolving after effective treatments: 20% mannitol 250 mL and dexamethasone 10 mg for 2 days, prophylactic antibiotics for one day, and an average of 3 days bed rest
		Open surgery performed before 2009 (*n* = 14) • Significant reductions in baseline mean pain NRS scores with low postoperative mean pain NRS scores 3.4 ± 2.5 • All symptoms and neurological deficits had been either completely or substantially resolved immediately after operation or during follow-up visits	• MRI-confirmed cyst recurrence (*n* = 3) with symptom recurrence (*n* = 3), one received a second operation with no symptom improvement and two others refused a second surgery for unknown reasons • Complications (*n* = 3, 21.4%) • CSF leakage (*n* = 3), all undergoing an artificial dural patch in a second operation and postoperative lumbar drainage for about one week
		Conservative management (*n* = 12) • Baseline mean pain VAS scores remained unchanged at follow-up, 5.8 ± 2.1 to 6.1 ± 2.2 • Symptoms were aggravated over time in 9 patients • Three patients achieved substantial relief of preoperative symptoms and neurological deficits	• Treatment failure (75%) • Cysts persisted with MRI follow-up

*NR* not reported

^+^Jiang et al. treatment recovery categories: *excellent* (returning to regular employment without any signs or symptoms), *good* (partial resolution of symptoms that did not interfere with return to work), *fair* (no improvement in pain or function but shrinkage of cyst), and *poor* recovery (no improvement in symptoms or cyst shrinkage)

^++^Murphy et al. treatment success categories: *excellent outcome* (complete pain relief, discontinuation of all pain medications, improvement or stabilization of cyst related neurological signs and symptoms, not requiring further treatment, satisfaction with results, and willingness to undergo another procedure if required, *good/satisfactory* outcome (pain improved on the Lumbar Spine Outcomes Questionnaire scale, discontinuation of narcotic analgesics, neurologic deficits commensurate with adequate function or no further progression, satisfied with treatment results, and not seeking further treatment. *Treatment failures* included all outcomes other than excellent or good/satisfactory even if some improvement was noted

Treatment failure rates ranged from 14 [96] to 25% [44]. Minor procedure-related transient side effects such as nausea, vomiting, low-grade fevers, cutaneous allergic reactions, and headaches were noted for patients undergoing these procedures (Table 3). However, in an early report by Patel et al. [97], three unusual cases of low-grade fevers and meningism believed to be aseptic meningitis occurred and were managed conservatively. The condition was also later referred to as chemical meningitis for a similar event in a surgical study for symptomatic Tarlov cysts [99]. Although the authors had not performed myelography to assess communication of the cyst with the subarachnoid space, it is thought that the treated cysts were likely wide-necked cysts. At that time, a wide-necked cyst was an unknown contraindication to percutaneous fibrin sealant injections as there is an increased possibility of fibrin sealant migration into the subarachnoid space. No cases of aseptic meningitis have been reported in any other percutaneous fibrin sealant injection studies including the large cohort of Murphy et al. [100]. In their study, procedures were performed under CT fluoroscopic guidance, employing a two-needle technique, and commercially prepared fibrin sealant; wide-necked cysts were a contradiction for their study.

Murphy et al.’s [74] 213-patient cohort undergoing percutaneous cyst aspiration and fibrin-sealant injection for symptomatic sacral Tarlov cysts is the largest study for any intervention performed for these cysts to date and includes 5-to-10-year long-term follow-up. As Tarlov cysts are not always associated with presenting symptoms, several diagnostic studies including lidocaine/or marcaine injection and cyst aspiration were performed in the study in order to determine if presenting symptoms could be attributed to the cyst. Pending resolution of symptoms, aspiration of cyst fluid, and fibrin sealant injection were performed.

**Fig. 10:**
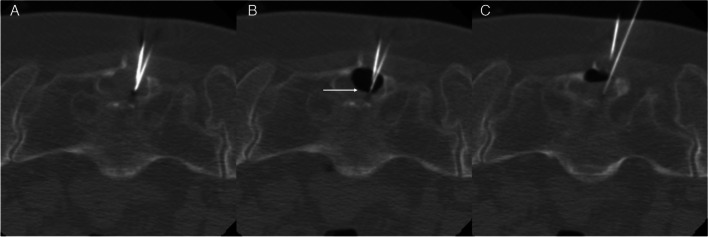
Two-needle technique for therapeutic aspiration and fibrin sealant injection of a symptomatic right S3 Tarlov cyst. **A** Two 22G spinal needles were advanced into the cyst under CT fluoroscopic guidance with one needle placed deep and the other more superficially. The stylets were then removed. **B** Aspiration from the needle placed in the deep aspect of the cyst was performed which produced a stable air-fluid level within the cyst (arrow). **C** Fibrin sealant (Tisseel VH; Baxter Healthcare, Westlake Village, California) was then injected into the cyst, with the volume injected equating to 80% of the aspirate volume

**Fig. 11:**
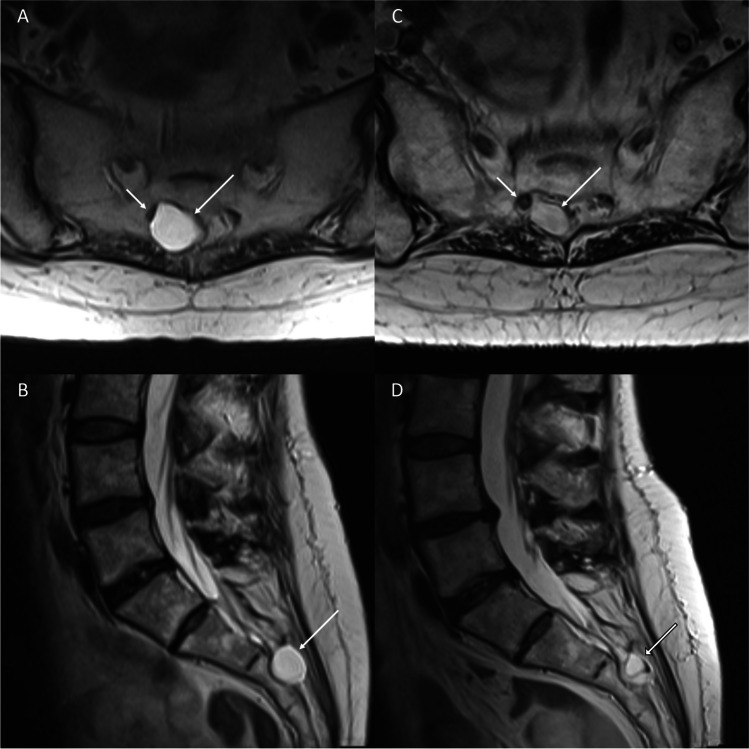
T2-weighted axial and sagittal images of a symptomatic right S3 Tarlov cyst belonging to the patient in Fig. 9, before and after fibrin sealant injection. **A**, **B** Preprocedural T2-weighted images show a large Tarlov cyst in the remodelled right S3 foramen (arrow) which compresses the right S3 nerve (short arrow). **C**, **D** Follow-up imaging performed at 32 months demonstrates lasting collapse of the cyst (arrow) with resolution of neural compression of the exiting S3 nerve which assumes a rounded morphology (short arrow)

The technique for the outpatient procedure was fully described by Murphy et al. [81]. A technical refinement using a two-needle approach (Fig. 10) was detailed in an earlier report and has proven useful for draining any closed space [100]. The technique involves positioning one needle deep in the cyst through which aspiration was performed, while the second needle placed more superficially acts as a venting tube during the aspiration allowing air to enter the cyst and function as a contrast agent. Needles are advanced into the cyst under CT fluoroscopic guidance, and as the cyst wall is penetrated, the patient may experience sharp pain reproducing the symptoms providing confirmation that the correct lesion is being treated. After confirming a stable intracystic fluid level, commercially available fibrin sealant is injected into the cyst through the deep needle with careful monitoring of the fill level with 80% of the cyst filled with fibrin. Follow-up MR imaging can demonstrate complete or partial collapse of the cyst with resolution of compression of the accompanying sacral nerve (Fig. 11).

In that study [74] of 421 patients with imaging confirmed sacral Tarlov cysts, 8.7% (34 patients) were referred for invasive surgery and 31 of them achieved excellent or satisfactory results. Also, eleven patients initially failing cyst aspiration also all achieved successful relief of symptoms with surgery. In the 213 patients undergoing aspiration-fibrin sealant injections, overall treatment success based on four criteria was rated as excellent or good in 81.8%, and at 1 year, 81% were satisfied with treatment outcomes (Table 3). Treatment success rated as excellent or good was 70% or greater for multiple individual presenting symptoms and neurological deficits.

Although most reports on cyst aspiration-fibrin sealant treatment for symptomatic Tarlov cysts involved single cohort studies, Jiang et al.’s [95] observational study compared three different treatment approaches over time (Table 3). Among them are conservative management (physiotherapy, anti-inflammatory, and neuropathic medications) for those refusing surgery (*n* = 12), open surgery involving partial cyst wall fenestration and imbrication followed by 2-day lumbar CSF drainage performed before 2009 (*n* = 14), and aspiration-fibrin sealant injection performed after 2009 (*n* = 56). Patients in the surgical and interventional group both had significant reductions in mean pain NRS scores, although post-operative pain scores were lower in the interventional than the surgical group (1.3 ± 1.1 vs 3.4 ± 2.5; *P* < 0.001).

In the surgical group, three patients had recurrent symptoms and MRI confirmed cyst recurrence, and in the interventional group, no recurrences occurred. Adverse events involving CSF leakage occurred in three surgical patients requiring a second operation whereas no post-operative infections, nerve damage, or CSF leaks occurred in the interventional group. In the medical management group, mean pain scores did not improve post-operatively, and in nine of the 12 patients, pain was aggravated.

### Complications

Percutaneous aspiration-fibrin sealant injection procedures are performed on an outpatient basis under conscious sedation and analgesia with patients discharged the same day. In a recent systematic review [101] of six studies (417 patients) of percutaneous aspiration-fibrin sealant procedures, commonly reported adverse events included allergic response to fibrin sealant (3.4%, 14 patients), transient post-operative sciatica (5.3%, 22 patients), and CSF leaks (1.7%, 7 patients) occurring in one study [74]. As discussed earlier, three unusual cases of low-grade fevers and meningism believed to be aseptic meningitis occurred following percutaneous fibrin-sealant injection [97], but have not been reported since.

Recurrence is possible after fibrin sealant injection as the material can gradually break down over time allowing for cyst recurrence. In the largest cohort study of aspiration-fibrin sealant injection for Tarlov sacral cysts and the longest follow-up, 36 patients (16.9%) had symptom recurrence, 23 patients within 6 months, and 13 patients after 6 months [74]. Fluid re-accumulation in the cysts were demonstrated on MR imaging, and all underwent re-aspiration and fibrin sealant injection, and all except one resulted in satisfactory symptom relief.

### Surgical management

Surgical objectives for Tarlov cysts are generally to relieve nerve compression and/or stimulation, stop bone erosion, and relieve symptoms. However, the sacral region is a very surgically technically demanding area. Cysts are mainly located in sacral areas where there is increased risk of ectasia and CSF leakage. Cyst walls are often fragile and can have adhesions. Even microsurgical approaches to reduce cyst size have the potential to damage nerve roots within or near the cyst, and it is difficult to repair any anatomy.

Initially, a variety of shunts were employed to address symptomatic Tarlov cysts by relieving or equalizing CSF pressure—lumboperitoneal shunt [102] and cyst-subarachnoid shunt [103, 104]. The results of these simple sacral decompression methods by diverting CSF, similar to percutaneous fluid aspiration alone, were not always effective or long lasting. In addition, shunts posed potential risks of malfunction, migration, and infection [105, 106]. Open surgical approaches involving simple sacral bony decompression have also been largely found to be inadequate due to low clinical success [107].

There is no consensus on the optimal surgical method for Tarlov cysts, and there have been numerous evolving surgical techniques [17, 18, 108–112]. Laminectomy or laminoplasty to unroof the sacral canal is commonly followed by varying microneurosurgical techniques to aspirate CSF, decrease cyst size, block communication between the cyst and the subarachnoid space to prevent CSF re-accumulation, and close the wound [17, 108, 113, 114].

Reports on microsurgical strategies to decrease cyst size after opening the dural sac and draining cyst CSF have involved: partial cyst resection, full cyst resection, or cyst fenestration. Depending on the cyst size, either imbrication involving resection of excess cyst wall prior to suture closure, or plication involving folding the cyst wall upon itself without resection prior to suture closure have been employed. In addition, various materials, gelfoam, fat or muscle grafts, and fibrin sealant have been used to fill the cyst cavity, block communication between the cyst and subarachnoid space, and cover dural defects. Simple resection of cyst wall or clipping are less commonly practiced, as they are considered a hazard to the sacral nerves within the cyst [18]. A cyst wall fenestration rather than cyst wall resection has been recommended to avoid or minimize any damage to the neural elements lying along the cyst wall [17, 115].

Because of the risk of neural damage with any handling of the cyst wall, several investigators report performing the surgery under electrophysiological monitoring [14, 111, 113, 114, 116]. Some investigators have also recommended placing lumbar drains post-operatively to address CSF leaks for variable periods of time (3 to 10 days) particularly for large cysts, large dural defects, or unsecure closures [108, 111, 116, 117]. Others, however, have reported that postoperative drains were not placed or were considered not appropriate [17, 106, 118]. Surgeries are performed as in-patient procedures, and hospital stays depending on bed rest policies and placement of CSF drains under general anesthesia have been variably reported: 3.5 days [107], 4 days (range 1–10 days) [99], 7 days (range 3–16 days) [113], 10 days (range 5–14 days) [118], and 15 days [110].

The majority of surgical reports for symptomatic Tarlov cysts involved single cohort studies; there have been three comparative studies of surgical techniques, none involving randomization [99, 119, 120]. The results of these comparative studies are detailed in Table 4. The studies involved a comparison of cyst fenestration versus nerve root imbrication [120], surgical approaches for cysts with or without spinal nerve root fibers [119], and cyst fenestration and nerve root imbrication versus cyst fenestration and partial cyst wall removal [99]. In all studies, patients had diverse symptoms and neurological deficits, and treatment outcomes were assessed differently (MacNab, IJOA criteria). Except for the Medani et al. [99] study, most patients in either group achieved successful resolution of various pain symptoms and neurological deficits; bowel and bladder dysfunctions however were less likely to improve. Adverse events, particularly CSF leaks occurred in all studies and was reported to be significantly higher in the fenestration than the imbrication group (42% vs 22%) [99].

**Table 4:** Comparative surgical procedures for symptomatic Tarlov cysts

Author, report year, country	Study cohort	Study design and interventions	Treatment outcomes
Xu [120] 2012 USA	• 15-patient cohort (6 women, 9 men) mean age 37.8 years (range 23 to 60 years) • Between 1998 and 2010 treated MRI documented symptomatic sacral Tarlov cysts • Symptoms included: low back pain or sacrococcygodynia (*n* = 12), sacral radiculopathy (*n* = 7), numbness (*n* = 6), sensory disturbance of the sacral dermatome (*n* = 9), claudication (*n* = 4), and bowel and bladder dysfunction (*n* = 6) • Eligible criteria included cyst diameter more than 1.5 cm, neurological symptoms and signs attributed to sacral Tarlov cysts serious enough to warrant treatment, no or little response to medical and physical therapy and a positive trial of CT guided aspiration • Tarlov cysts were surgically treated differently before and after 2006	• Prior to 2006, first surgery group included 6 patients (1 woman, 5 men) age range 23–52 years • Surgical procedure included sacral laminectomy, microsurgical cyst fenestration and cyst wall imbrication with free autologous fat or muscle grafts over the closed wall • Follow-up ranged from 13 to 124 months	• In the first surgery group, patients experienced either complete remission (*n* = 3) or substantial relief (*n* = 2)) of symptoms and neurological deficits immediately after surgery or during follow-up visits • Symptom and cyst recurrence (*n* = 1) without improvement with second surgery • Adverse events (*n* = 1), CSF leak fixed with artificial dural patch in second surgery and one week lumbar drainage
		• Since 2006, second surgery group included 7 patients (3 women, 4 men) age range 23–59 years • Surgical procedure included sacral laminectomies, after which the cyst wall was partially removed and fenestrated with a scalpel to drain CSF fluid and the CSF leak aperture was located and repaired with fat and fibrin sealant • Follow-up ranged from 14 to 61 months • Two patients, both women, refusing surgery underwent medical management including analgesics, nonsteroidal anti-inflammatories and physical therapy, followed for 50 and 62 months	• In second surgery group, six patients experienced complete remission (*n* = 5) or substantial relief (*n* = 2) of symptoms and neurological deficits immediately after surgery or during follow-up visits • There were no recurrences • Adverse events (*n* = 1) with worsening of preoperative bladder dysfunction, gradually returning to normal one month later • No neurological deficits, CSF leaks,or surgical infections, postoperative lumbar drains were not placed • Patients in the medical management group (*n* = 2) experienced aggravated symptoms and increased cyst growth over 4-year follow-up
Sun [119] 2013 China	• Between 2009 and 2012 consecutive 55 patient cohort (38 women, 17 men), mean age 40.4 ± 14.31 years (range 13 to 70 years) with mean follow-up 25.5 ± 12.6 months • Symptoms were present at multiple locations (40%) with the most common symptoms: pain (73%), numbness (9.1%), bowel/ bladder and sexual dysfunction (3.6%), lower extremity weakness (1.8%)and tenesmus (1.8%) with symptom duration of 39.8 ± 51.55 months • Cysts maximum diameter > 1.5 cm, mean number of cysts 1.5 ± 0.72 • Eligibility criteria included radiological findings consistent with Tarlov cysts and neurological symptoms attributable to cysts • Tarlov cysts were surgically treated differently for sacral cysts having (*n* = 34) and not having (*n* = 21) spinal nerve root fibers within the cyst	• After laminectomy the terminal thecal sac was identified and dissected free from the overlying cysts and cysts were dissected from surrounding structures to reveal its origin and relationships with nerve fibers • Intraoperative neurophysiological monitoring was used to differentiate nerve root fibers from other tissues, electrical stimulation verified absence of motor nerve fibers, and closure was reinforced with a local muscle flap • Cysts without nerve root fibers tended to be significantly larger than cysts with nerve root fibers (4.9 cm ± 2.60 vs 3.3 cm ± 1.61) and tended to occur as single cysts (91% vs 47%)	• Mean duration of prone position after surgery was 4.9 ± 2.16 days, mean hospital LOS 15.8 ± 5.35 days • Mean post-operative IJOA cores (neurological function) significantly improved in both groups: cysts with nerves (18.9 ± 1.22 to 19.6 ± 0.59) vs cysts without nerves (17.7 ± 2.17 to 19.5 ± 1.03), without significant group differences • Lower extremity weakness improved in both groups (7/9 cases vs 8/9 cases), pre-operative sensation dysfunctions improved in all • Although the majority had normal pre-operative bowel/bladder function – 9/15 patients with bowel/bladder dysfunction preoperatively did not improve (4 cysts with nerves, 5 cysts without nerves) • Wound healing was classified as well healed (86%), delayed healing (7%), and 7% (4 case) required further debridement and resuturing with two of these patients requiring a second operation due to worsening pseudomeningocoele 2 months post-operatively • Cyst resolution was complete (55%), small residual cyst (16%) and cyst disappearance but large effusion into the canal cavity (29%)
		• For cysts without nerve fibers (*n* = 21), the neck of the cyst was transfixed, ligated and the remaining cyst wall resected distal to the ligation. If the cysts were associated with a tethered cord, then untethering was performed during the same procedure	
		• For cysts with nerve fibers (*n* = 34), cysts were partially resected, defects oversewn to prevent CSF leakage from the subarachnoid space, nerve root sheath reconstructed, and redundant cyst wall shrunk using bipolar cautery	
Medani [99] 2019 United States	• Between 2007 and 2013 consecutive 36-patient cohort (31 women, 5 men), mean age 51 years (range 26–84 years) • Symptoms included low back pain (*n* = 34), sensory dysfunction (*n* = 25), bladder and/or bowel dysfunction (*n* = 22), dyspareunia (*n* = 4), erectile dysfunction (*n* = 2) • Tarlov cysts were surgically treated either by simple cyst fenestration (*n* = 12) or nerve root imbrication (nerve root repair) (*n* = 27)	• Cyst fenestration surgeries from 2007 to 2009 (*n* = 11, 1 redo) • Surgeries involved partial sacral laminectomy, cyst fenestration, and injection of fibrin glue into the cyst lumen • Procedures for 2 or more cysts were performed for 6 fenestration procedures • Overall, operative microscope was used in 8 surgeries, intraoperative electromyographic monitoring used in all 12 surgeries • Lumbar drain placed postoperatively in 4 procedures, median hospital LOS 4 days (range 1–15 days)	• Symptomatic improvements at all follow-up points (post-operative, 3, 12, and 24 months) were rated more often in modified ^+^MacNab poor/fair categories (*n* = 9) than good/excellent (*n* = 3) at 3 months • Surgical related complication rate 42% (*n* = 5) • Adverse events included; contained CSF leak or pseudomeningocele (*n* = 4), wound infection (*n* = 1), • Redo procedure for no improvement or worsening of symptoms (*n* = 1) • No symptomatic cyst recurrence
		• Nerve root imbrication (nerve root repair) from 2010 to 2013 (*n* = 25, 2 redo) • Procedures for 2 or more cysts were performed for 12 imbrication procedures • Imbrication surgeries involved sacral laminoplasty, nerve root imbrication using non-absorbable materials (usually Nylon 6–0) — fibrin glue was injected around reconstructed nerve root in 18 procedures • Overall, operative microscope was used in 25 procedures, intraoperative electromyographic monitoring used in 25 procedures • Lumbar drains placed in 15 procedures, median hospital LOS 4 days (range 1–10 days)	• Symptomatic improvements at all follow-up points (post-operative, 3, 12, and 24 months) were rated more often in modified ^+^MacNab poor/fair categories (*n* = 14) than good/excellent (*n* = 7) at 3 months • Surgical related complication rate 22% (*n* = 6) • Adverse events included contained CSF leak or pseudomeningocele (*n* = 4), wound dehiscence (*n* = 1), chemical meningitis (*n* = 1) • Redo procedure for no improvement or worsening of symptoms (*n* = 2) • Symptomatic cyst recurrence requiring intervention (*n* = 4)

*CSF* cerebral spinal fluid, *LOS* length of stay, *IJOA* Improved Japanese Orthopaedic Association, *LOS* length of stay

^+^MacNab criteria: *excellent* (no pain, no restriction of mobility, return to normal work and level of activity); *good* (occasional non-radicular pain, relief of presenting symptoms, able to return to modified work); *fair* (some improved functional capacity, still handicapped and/or unemployed); *poor* (continued objective of nerve root involvement, additional operative intervention needed at index level irrespective of length of postoperative follow-up)

More evidence on surgical procedures for symptomatic Tarlov cysts is available from several systematic reviews by authors covering different time periods: Lucantoni et al. up to 2011 [105], Dowsett et al. up to January 2016 [121], Sharma et al. up to April 2018 [101], and Kameda-Smith et al. to April 2019 [122]. However, two of the reviewers [105, 121] included other non-surgical procedures, such as percutaneous aspiration-fibrin sealant interventions, epidural steroid injections, and endoscopic procedures in the pooled summaries of surgeries limiting the usefulness of the reviews.

Summaries of Kameda-Smith’s [122] and Sharma’s [101] systematic reviews on open surgery are detailed in Table 5. Both reviews included meta-analyzed outcomes in surgical management of symptomatic Tarlov cysts—16 studies (283 patients), excluding those with less than 10 patients in Kameda-Smith et al.’s review [122] and 32 studies (333 patients) in Sharma et al.’s review [101]. The mean age of patients in the reviews was 46 ± 8.6 years and 45 ± 13 years with females predominating (70%, 71%). Presenting symptoms and neurological deficits at baseline were diverse and long lasting with mean duration 40 ± 26 months [122] (Table 5).

**Table 5:** Systematic reviews of open surgical and percutaneous interventions for symptomatic Tarlov cysts

Author, number of studies reviewed	Presenting demographics and symptoms	Cyst information	Treatment outcomes (meta-analyzed events)	Adverse events (meta-analyzed events)
Kameda- [122] 2021 Systematic review 16 studies (283 patients) open surgical management	• Mean age 46 ± 8.6 years, 71% women • Mean 27.4 ± 11.5 month follow-up • Symptoms with Tarlov cysts included lower back pain (45%), lower extremity pain (52%), perineal/perianal pain (24%), motor deficits (16%), sensory deficits (25%) urinary or bladder dysfunction (46%), and sexual dysfunctions (6%) • Pre-operative symptom duration 40 ± 26 days	• Multiple or bilateral cysts occurred in 42% patients • Mean cyst size 3.0 ± 1.0 cm	• Complete or partial symptom resolution 81% (95% CI, 74–88%) • Complete or substantial reduction in cyst size 79% (95% CI, 42–99%) • Symptoms decreased post-operatively in motor deficits (17.8 to 5.4%), sensory deficits (47 to 14.5%) and bowel dysfunction and urinary incontinence (40 to 14.3%) • Symptom recurrence 8.3% (95% CI, 2.7–6.3%) • Cyst recurrence 8.5% (95% CI, 3.5 –15.4%)	• Overall surgical complication rate 16.9% (95% CI, 11.8–22.7%) ranging from 5.6 to 31.4% • Adverse events: CSF leaks 4.8%, surgical site infections 4.3% (95% CI, 2.4–8.1%), and new or worsened bladder dysfunction 2.1% (95% CI, 0.07–4.0%) • Re-operation rate 6.7% (95% CI, 2.9–12%)
Sharma [101] 2019 systematic review 32 studies (333 patients) open surgical management	• Mean age 45 ± 13 years (range 21–83 years), 71.4% women • Mean 38 ± 29 month follow-up • Symptoms with Tarlov cysts included; back pain 82.8%, sacral radiculopathy 51.4%, bowel incontinence 20.3% and bladder incontinence 37.8% • Other symptoms occurring less frequently than in percutaneous group studies: coccygogynia (25.5% vs 65.8%), perineal pain (26.5% vs 77.6%), lower limb weakness (11.7% vs 36.3%), sensory disturbances (35.7% vs 62.7%), and sexual dysfunction (4.6% vs 31.2%)	• Cysts occurred as solitary cysts (*n* = 122) and multiple cysts (n = 82) • Cysts were commonly located at the S1–S3 sacral levels • Cyst size ranged from 0.8 to 10 cm	• Symptomatic improvement, 83.5% • Symptom recurrence, 21% (95% CI, 12–54%) • Cyst recurrence, 8% (95% CI, 5–10%)	• Overall surgical complication rate 21% • Adverse events: CSF-related complications (CSF leaks, fistula, pseudomenigocele) (9%), transient sciatica 17% (95% CI, 4–30%)], sexual dysfunction 11% (95% CI, 0 – 21%), bladder and bowel complications (sphincter weakness, urinary incontinence and overflow incontinence) 12% (95% CI, 8–15%), and wound infection requiring debridement and extended hospital stay with external CSF drainage 5%, (95% CI, 4–7%) • Other complications 18% (95% CI 9–26%) included marked venous bleeding, transient intracranial hypotension, superficial seroma, incisional erythema, cerebellar intracerebral hemorrhage, prostatitis
Sharma [101] 2019 Systematic review 6 studies (417 patients) percutaneous cyst aspiration and fibrin sealant injection	• Mean age 38 ± 10 years (range 20–73 years), 74% women with mean follow-up 15 ± 12 months • Symptoms with Tarlov cysts included; back pain, 82.8%, sacral radiculopathy 51.4%, bowel incontinence 20.3% and bladder incontinence 37.8% • Other symptoms occurring more frequently than in surgical group studies: coccygogynia (65.8% vs 25.5%), perineal pain (77.6% vs 26.5%), lower limb weakness (36.3% vs 11.7%), sensory disturbances (62.7% vs 35.7%), and sexual dysfunction (31.2% vs 4.6%)	• Cysts occurred as solitary cysts (*n* = 82) and multiple cysts (*n* = 122) • Cysts were commonly located at the S2-S3 sacral levels (*n* = 264) • Cyst size ranged from 1.6 to 3.2 cm	• Symptomatic improvement 83.5% • Symptom recurrence 20% (95% CI, 10–50%) • Cyst recurrence 20% (95% CI, 10–50%)	• Overall complication rate 12.5% • Adverse events: CSF leak 3% (95% CI, 1–5%), transient sciatica 8% (95% CI, 24–39%), bowel/bladder 1% (95% CI, 1–3%) • Other complications 3% (95% CI, 5–12%) included allergic reactions to sealants

Surgeries in both reviews involved multiple techniques, laminectomies or laminotomies followed by microsurgical cyst resection, partial resection, fenestration, imbrication, and plication with lumbar drains placed infrequently. Various materials including fat grafts, muscle grafts, gelfoam, or fibrin sealant were used in surgeries to reinforce cyst closure. The presence of bony defects or erosions required additional procedures. Heterogeneity of the surgical approaches, variability in institutional/operational protocols, inconsistent reporting, and retrospective nature of studies were limitations cited in both reviews.

High rates of treatment success with variable definitions were reported in both reviews for operative management of cysts. Kameda et al. [122] reported complete or partial symptom resolution in 81% (95% CI, 74–88%) with 79% (95% CI, 42–99%) also having a complete or substantial reduction in cyst size. Sharma et al. [101] reported an 83.5% overall symptomatic improvement.

Although cyst recurrence was similar in the two reviews, 8.5% (95% CI, 3.5–15.4%) [122] and 8% (95% CI, 5–10%) [101], symptom recurrence at 21% (95% CI, 12–54%) was higher in the Sharma et al. [101] review than the 8.3% (95% CI, 2.7–16.3%) in the Kameda et al. [122] review. A re-operation rate reported in one review [122] was 6.7% (95% CI, 2.9–12%).

### Complications

Kameda et al. [122] reported an overall complication rate of 16.9% (95% CI, 12–23%) ranging from 5.6 [114] to 31.4% [113]. The main complications included CSF leaks 4.8%, surgical site infections 4.3% (95% CI, 2.4–8.1%), and new or worsened bladder dysfunction 2.1% (95% CI, 0.07–4.0%). Sharma et al. [101] reported a 21% overall complication rate for the surgical group. Complications in that review included CSF-related complications (CSF leaks, fistula, pseudomenigocele) 9%, sexual dysfunctions 11%, bladder and bowel complications 12%, and wound infections requiring debridement and extended hospital stay with external CSF drainage 5%. Complications were thought to be mainly related either to the inadequate closure of the dura and/or handling sacral nerve roots.

### Open surgery versus percutaneous aspiration-fibrin sealant injection

Although there have been no randomized trials of open surgery versus percutaneous aspiration-fibrin sealant injection, Sharma et al.’s [101] review made a comparison between studies (32 studies, 333 patients) of open surgical management with studies (6 studies, 417 patients) on aspiration-fibrin sealant interventions for symptomatic Tarlov cysts. However, one of the percutaneous studies [5] included only cyst fluid aspiration, and the others [46, 74, 96, 97, 123] included both cyst aspiration and fibrin sealant injection. Cyst aspiration only techniques are now usually intended mainly as diagnostic studies as symptoms and cysts usually recur without injection of fibrin sealant.

In both treatment groups, women predominated in the pooled surgical (71.4%) and percutaneous (74%) groups,although patients in the percutaneous group tended to be younger than the surgical group (38 ± 10 years vs 45 ± 13 years). Cyst size also tended to be smaller in the percutaneous group versus the surgical group (range 1.6 to 3.2 cm vs 0.8 to 10 cm) and were commonly located at the S2–S3 sacral level rather than the S1–S3 sacral level in the surgical group. Diverse presenting symptoms of pain and neurological dysfunctions were frequently reported in both study groups although higher incidences of several symptoms were reported for the interventional group; coccygodynia, perineal pain, lower limb weakness, sensory disturbances, and sexual dysfunction (Table 5).

Symptomatic improvement was 83.5% in both treatment groups although transient exacerbation of symptoms was greater in the percutaneous group (10.1% vs 3.3%). Recurrence of symptoms was similar in the treatment groups (20% vs 21%), but cyst recurrence was higher in the percutaneous group (20% vs 8%). The high cyst recurrence rate in the percutaneous group was likely associated with the more than 2-year follow-up for recurrence in the Murphy et al. study [74].

However, the overall procedural-related complication rate was significantly higher for the surgical group versus the percutaneous group (21% vs 12.5%). As noted earlier, complications in the percutaneous study groups were mainly minor transient events such as transient sciatica or allergic reaction to fibrin sealant. In the surgical study groups, all adverse events were significantly higher than the percutaneous study groups: transient sciatica (17% vs 8%, *P* = 0.0177), CSF-related complications (CSF leaks, fistula, pseudomenigocele) (9% vs 3%, *P* = 0.017), sexual dysfunction (11% vs 0%, *P* = 0.0007), and wound infection requiring debridement and extended hospital stay with external CSF drainage (5% vs 0%). Bowel and bladder complications were also higher for the surgery groups (12% vs 1%, *P* = 0.0007) and are concerning as these may be irreversible occurrences.

### Treatment overview

There is no agreed upon optimal surgical treatment for patients with symptomatic Tarlov cysts, and the numerous evolving surgical techniques tend to support a lack of consensus [17, 18, 108–112]. Treatment considerations for these cases are complex and best based on a case-by-case basis depending on a variety of factors including the patients’ health status, characteristics of the sacral cyst, and adverse impacts in the sacral region and elsewhere.

It is worth noting that women constituted 70% of the study groups treated either percutaneously or surgically for Tarlov cysts and that they often presented with years of long-standing debilitating pain and neurological dysfunctions. Several authors have proposed that inadequate knowledge due to the rarity of these cysts or gender bias by treating physicians contributes either to a significantly delayed or a lack of treatment for these patients [45, 46].

A key treatment consideration for providers and patients is to determine whether to consider minimally invasive percutaneous or open surgical interventions for symptomatic Tarlov cysts. There is sufficient evidence supporting both minimally invasive percutaneous fibrin sealant procedures and open surgical interventions for effective treatment of symptomatic Tarlov cysts. However, due to the rarity of these cysts, the evidence is largely based on small cohort studies, except for one study [74]. There have been no randomized trials between these interventions although several systematic reviews have shown that both approaches have similarly high effectiveness in reducing Tarlov cyst-associated symptoms.

Post-operative management and recovery, however, are significantly different after these procedures. Following percutaneous aspiration-fibrin sealant interventions, patients are usually discharged the same day with minimal restrictions. After surgery, patients are often kept prone for several days to control CSF pressures and/or place external lumbar drains, and hospital stays, when reported, often involve lengthy durations variably reported from 3 to 15 days. Several systematic reviews [101, 122] reported that although symptom recurrence was similar for the treatment groups, cyst recurrence was significantly higher for percutaneous aspiration-fibrin sealant interventions than surgery. However, successful repeat percutaneous treatments for symptom recurrence have been reported [74], and these procedures are likely to be more easily performed than repeat open surgical procedures. Furthermore, patients failing an initial percutaneous treatment were able to successfully undergo further invasive sacral interventions if appropriate [74].

Complication rates in systematic reviews have been reported to be significantly higher after surgical interventions than percutaneous aspiration-fibrin sealant procedures [101, 122]. The main complication in the percutaneous group was allergic reactions which are rare events and transient sciatica which also occurred after surgery and at a higher rate. Complication rates are also highly variable in the surgical studies due to the extensive array of open surgical laminectomies/laminotomies with variable microsurgical approaches to managing the sacral cyst, CSF fluid, and wound closure. Depending on the surgical approach, manipulations of the cyst wall in which nerve fibers run through increases the potential for neurological damage, some being irreversible.

However, some cysts depending on their size, distribution, and damage or erosion to sacral bony areas could be better approached by surgery. Cysts having a wide neck or a large communication pore with the subarachnoid space would be a contraindication to fibrin sealant injection and would also be likely better treated surgically. For both percutaneous fibrin sealant procedures and open surgeries, the lengthy delay to treatment is a significant concern particularly as the neurological deficits occurring from nerve fiber neuropathy caused by Tarlov cysts may result in lengthy recovery periods or may become unrecoverable without an early intervention to ensure better neurological outcomes.

## Recommendations

**Fig. 12:**
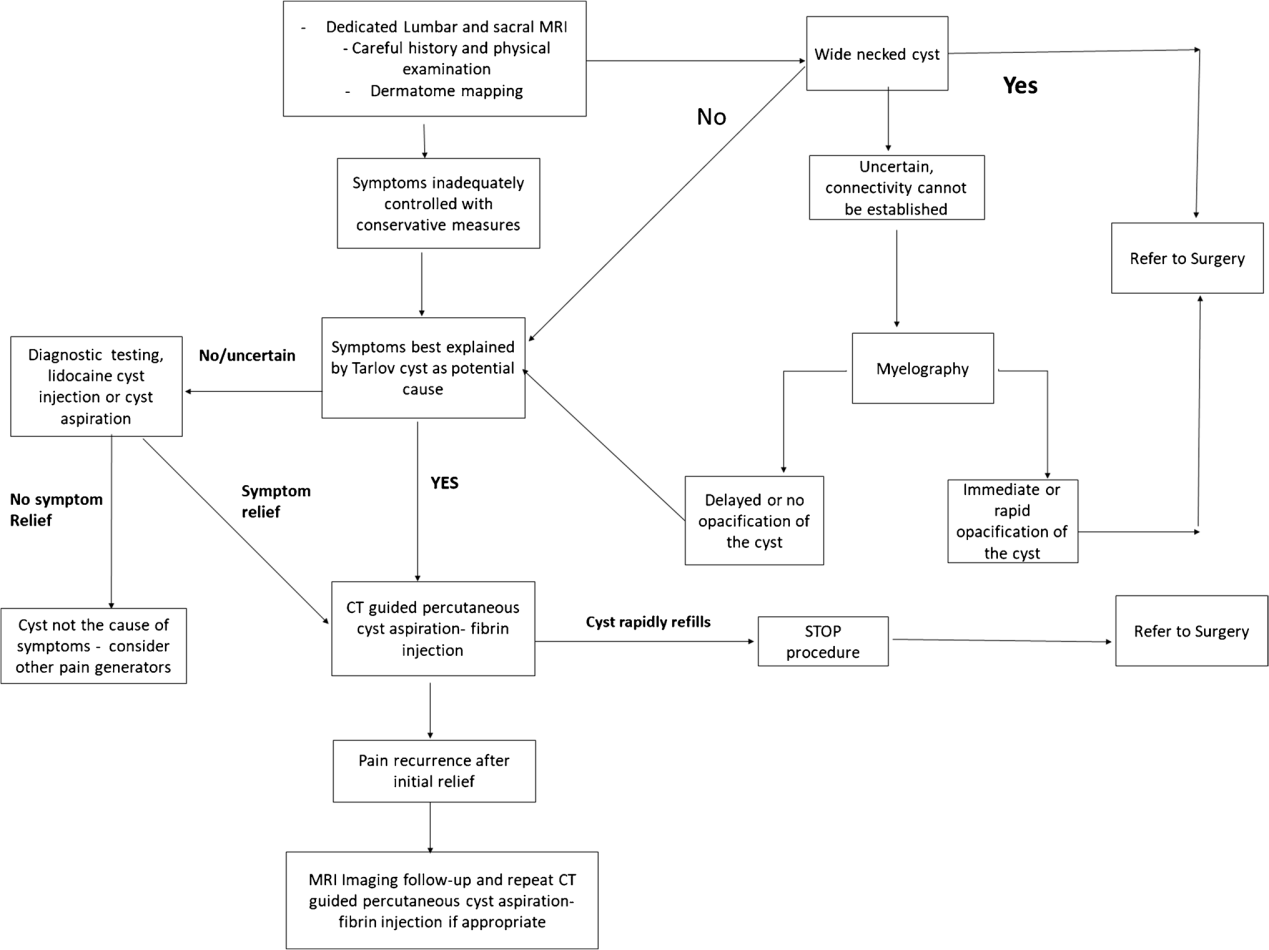
Treatment decision algorithm for symptomatic MRI-documented Tarlov cyst

Based on our experience over 1000 patient referrals and treatment experience with cyst aspiration-fibrin sealant injections of symptomatic Tarlov cysts and on a review of the literature on these cysts, we can make several key observations and recommendations on the management of these cysts. We also constructed a treatment decision algorithm to guide management of patients with symptomatic Tarlov cysts (Fig. 12).

1. Although sacral Tarlov cysts are an uncommon finding, prevalence estimates have often been based on incidental radiographic findings and inconsistent reporting particularly from lumbar rather than dedicated sacral MRIs and likely contribute to an underestimation of the prevalence of this spinal disease.
2. There is extensive evidence that sacral Tarlov cysts are associated with a diverse range of pain and neurological symptoms. Workups of patients, particularly for women presenting initially with low back pain or coccygodynial pain, should include a careful history, neurological examination, and when appropriate electrodiagnostic testing or urodynamic studies to conduct more comprehensive multidisciplinary investigations.
3. Imaging investigations for Tarlov cysts should include a lumbar spine MRI and a sacral MRI with axial and sagittal planes of the entire sacrum and when identified radiologically should be reported in a differential diagnosis and in the appropriate clinical context, considered a potential pain generator and contributor to neurological symptoms.
4. A range of strategies can be employed to determine if sacral cysts are causative or contributing agents of symptoms: Tarlov disease specific quality-of-life questionnaires, patient-completed dermatome maps, and diagnostic tests including percutaneous anesthetic injection into the cyst or cyst fluid aspiration with a two-needle technique. Fluid aspiration can also assess whether rapid cyst refilling occurs indicating a wide-necked cyst, a contraindication to fibrin sealant injection.
5. Sacral Tarlov cysts can cause peripheral nerve fiber neuropathy through sacral nerve root stretching or compression, and electrodiagnostic tests can detect nerve conduction abnormalities and nerve fiber neuropathy, potentially responsible for a range of commonly reported Tarlov related pain, paresthesia and, bowel/bladder dysfunctions. Early intervention for nerve fiber neuropathy is preferred to ensure better neurological outcomes. As patients with Tarlov cysts have often reported experiencing symptoms for years, there is a concern that long-standing nerve damage may be unrecoverable.
6. Both percutaneous and open surgical approaches have resulted in high rates of rapid symptomatic relief, but percutaneous interventions have several advantages over open surgery. The cyst aspiration-fibrin injection is an uncomplicated technique performed as an outpatient procedure with rapid reductions in symptoms and recovery with no serious complications. In comparison, there is no consensus on the optimal surgical method, and surgeries are technically demanding. They also involve variable approaches with significantly higher complication rates, longer hospital stays, and recovery than percutaneous aspiration-fibrin sealant interventions.

## Conclusion

Sacral Tarlov cysts are an uncommon spinal column disease that has been associated with a wide range of debilitating pain, neurological disturbances, and dysfunctions. The condition is often overlooked or ignored as an incidental finding of no clinical significance, particularly in the presence of other comorbid spinal pathologies. Tarlov cysts when identified radiologically should be reported in a differential diagnosis and in the appropriate clinical context evaluated as a potential pain generator and contributor to neurological symptoms.

Excluding symptomatic patients from appropriate evaluation disproportionately deprives women of appropriate care by delaying a potentially life-altering minimally invasive treatment resulting in a debilitating condition unmanaged for years. Based on risk–benefit perspectives and the extensive reported complications of open surgery, percutaneous aspiration-fibrin sealant interventions should be considered first-line treatment for patients with symptomatic sacral Tarlov cysts, following confirmatory investigations that cysts are symptomatic.

## Data Availability

All data relevant to this review are referenced in the article.
